# Proteomic Tissue-Based Classifier for Early Prediction of Prostate Cancer Progression

**DOI:** 10.3390/cancers12051268

**Published:** 2020-05-17

**Authors:** Yuqian Gao, Yi-Ting Wang, Yongmei Chen, Hui Wang, Denise Young, Tujin Shi, Yingjie Song, Athena A. Schepmoes, Claire Kuo, Thomas L. Fillmore, Wei-Jun Qian, Richard D. Smith, Sudhir Srivastava, Jacob Kagan, Albert Dobi, Isabell A. Sesterhenn, Inger L. Rosner, Gyorgy Petrovics, Karin D. Rodland, Shiv Srivastava, Jennifer Cullen, Tao Liu

**Affiliations:** 1 Biological Sciences Division, Pacific Northwest National Laboratory, Richland, WA 99354, USA; yuqian.gao@pnnl.gov (Y.G.); yi-ting.wang@pnnl.gov (Y.-T.W.); huiwangjd@gmail.com (H.W.); tujin.shi@pnnl.gov (T.S.); athena.schepmoes@pnnl.gov (A.A.S.); thomas.fillmore@pnnl.gov (T.L.F.); weijun.qian@pnnl.gov (W.-J.Q.); dick.smith@pnnl.gov (R.D.S.); 2 Henry M. Jackson Foundation for the Advancement of Military Medicine, Bethesda, MD 20817, USA; cymclg@gmail.com (Y.C.); dyoung@cpdr.org (D.Y.); ysong@cpdr.org (Y.S.); ckuo@cpdr.org (C.K.); adobi@cpdr.org (A.D.); gpetrovics@cpdr.org (G.P.); 3 Center for Prostate Disease Research, John P. Murtha Cancer Center Research Program, Department of Surgery, Uniformed Services University of the Health Sciences and Walter Reed National Military Medical Center, Bethesda, MD 20814, USA; inger.l.rosner.mil@mail.mil (I.L.R.); shsr629@gmail.com (S.S.); 4 Cancer Biomarkers Research Group, Division of Cancer Prevention, National Cancer Institute, Bethesda, MD 20892, USA; srivasts@mail.nih.gov (S.S.); kaganj@mail.nih.gov (J.K.); 5 Joint Pathology Center, Silver Spring, MD 20910, USA; isabell.a.sesterhenn.civ@mail.mil; 6 Department of Cell, Developmental, and Cancer Biology, Oregon Health and Science University, Portland, OR 97201, USA; 7 Department of Population and Quantitative Health Sciences, Case Western Reserve University, Cleveland, OH 44106, USA

**Keywords:** biochemical recurrence, biomarkers, early detection, metastasis, prostate cancer, proteomics

## Abstract

Although ~40% of screen-detected prostate cancers (PCa) are indolent, advanced-stage PCa is a lethal disease with 5-year survival rates around 29%. Identification of biomarkers for early detection of aggressive disease is a key challenge. Starting with 52 candidate biomarkers, selected from existing PCa genomics datasets and known PCa driver genes, we used targeted mass spectrometry to quantify proteins that significantly differed in primary tumors from PCa patients treated with radical prostatectomy (RP) across three study outcomes: (i) metastasis ≥1-year post-RP, (ii) biochemical recurrence ≥1-year post-RP, and (iii) no progression after ≥10 years post-RP. Sixteen proteins that differed significantly in an initial set of 105 samples were evaluated in the entire cohort (n = 338). A five-protein classifier which combined FOLH1, KLK3, TGFB1, SPARC, and CAMKK2 with existing clinical and pathological standard of care variables demonstrated significant improvement in predicting distant metastasis, achieving an area under the receiver-operating characteristic curve of 0.92 (0.86, 0.99, *p* = 0.001) and a negative predictive value of 92% in the training/testing analysis. This classifier has the potential to stratify patients based on risk of aggressive, metastatic PCa that will require early intervention compared to low risk patients who could be managed through active surveillance.

## 1. Introduction

Prostate cancer (PCa) is the most commonly diagnosed cancer in men, except for superficial skin cancer, and the second leading cause of death due to cancer among American men [1,2]. Prostate cancer has a complex disease spectrum, ranging from clinically indolent to aggressive subtypes with a high degree of molecular and cellular heterogeneity [3,4,5,6,7]. In order to provide optimal personalized management of the disease, both the physician and the patient need to consider if the disease is likely or unlikely to progress based on biomarkers and imaging tests and to then select the best course of action, either active surveillance for benign and low risk tumor or treatment for a tumor that is likely to progress. Several relevant aspects of current clinical practice are suboptimal. Prostate cancer screening based on serum prostate-specific antigen (PSA) results in many false positives, biopsy complications, and overdiagnosis that ultimately leads to overtreatment [8,9]. In addition, conventional primary and repeat biopsies can be inaccurate and pose unnecessary risks. Application of multi parametric MRI (mpMRI) in the context of diagnosis and active surveillance improved risk stratification and the identification of target lesions [10], while increasing the overall rate of cancer detection in higher grade groups. Currently, combining mpMRI and conventional biopsies provides the highest detection rate [11,12].

Early detection of PCa in isolation is not sufficient to reduce mortality from the disease, as a large proportion of screen-detected lesions are indolent. There is a critical unmet need to distinguish early between indolent and aggressive PCa, so that the proper treatment can be selected while overtreatment of indolent disease can be avoided. Although recent decades have seen the development of several RNA-based panels directed at prognosis for PCa [5,13,14], none of these has yet received endorsement as the early marker of choice for future progression, and the genes/proteins represented in the various assays do not overlap. In addition to mRNA-based biomarker panels, recent efforts have also explored the possibility of using protein-based biomarkers in PCa, most focused on the identification and verification of differentially abundant proteins in case-control studies, including the development of urinary protein signatures for extracapsular PCa [15]. While promising, none of these assays has yet entered clinical practice or demonstrated a significant improvement in prognostic accuracy over more traditional methods of assessing risk.

In this study, we have focused on the identification of reliable early molecular markers capable of accurately predicting future aggressive disease progression. Our objective was to develop a panel of protein biomarkers, detectable in radical prostatectomy (RP) samples from men with organ-confined PCa, that would improve the ability to stratify patients for risk of progression, defined as either biochemical recurrence (BCR) after prostatectomy or distant metastasis (DM). The feasibility of generating protein expression data for low abundance proteins from formalin-fixed parafilm-embedded (FFPE) specimens from primary prostate tumors was demonstrated using an antibody-independent targeted proteomics analysis (high-pressure, high-resolution separations coupled with intelligent selection and multiplexing-selected reaction monitoring (PRISM-SRM)) developed by our team [16]. Differential protein abundance was then used to identify proteins associated with PCa aggressiveness. The predictive accuracy of a proteomic classifier in predicting local and distant cancer progression was validated in a cohort of men with long-term follow-up data and detailed clinical annotation. The addition of the proteomic classifier to the traditional, Standard of Care (SOC) variables was examined using training and testing analysis to determine the ability of the classifier to improve performance in the study cohort.

## 2. Results

### 2.1. Selection of Biomarker Candidates

The initial list of candidate markers included 151 genes that were selected based on the following criteria [17]: (1) have significant differential gene expression in prostate tumor versus normal comparison; (2) are regulated by androgen; (3) are associated with prognosis of prostate cancer; (4) are associated with the ETS family of transcription factors; (5) are commonly rearranged in prostate cancer; (6) are involved in prostate cancer cell invasion; (7) are associated with multiple malignancies; (8) or can distinguish prostate epithelial from stromal cells (Table S1). These 151 genes were incorporated into a NanoString Code Set, which was used to identify differential mRNA expression in a series of PCa samples across clinical outcomes. Based on the significance in differential mRNA expression [17] and the likelihood of detecting them at protein level, the original list of 151 was down-selected to 52 candidates for targeted proteomics measurements using PRISM-SRM.

### 2.2. Development of PRISM-SRM Assays and Targeted Proteomics Measurements

The application of our antibody-independent PRISM-SRM method, which utilizes offline chromatographic separation and “intelligent” fraction selection via monitoring the heavy isotope-labeled peptide internal standards [16], allows for much higher sample loading (e.g., 70-fold in the current study), highly effective peptide enrichment, and significantly reduced sample complexity that provided much higher sensitivity and is thus well suited for the detection of protein biomarker candidates in a broad concentration range. Using synthetic peptides with and without heavy isotope labeling of C-terminal lysine or arginine, highly sensitive, precise, and multiplex PRISM-SRM assays were developed in our laboratory using procedures we previously established (see Section 4). The details of the PRISM-SRM assays such as the selected peptide sequences, assay conditions, and limit of detection and quantitation as well as the final sample measurement results are provided in Tables S2–S7. The linearity and interference issues have been carefully evaluated and demonstrated in Figures S1 and S2, respectively.

As expected, compared to the regular LC-SRM which detected 21 proteins, the PRISM-SRM method allowed for detection of a much larger set of 42 proteins (Table S2). Ten of the original 52 protein markers were excluded from further studies due to poor detection sensitivity in the FFPE prostatectomy specimens.

There were 16 protein markers that demonstrated an initial statistically significant difference in expression distribution across the three study outcome groups (i.e., DM, BCR, and non-progression) in an initial analysis of a subset of the samples (n = 105, including 20 DM, 37 BCR, and 48 non-progression samples), including ANXA2, CAMKK2, CCND1, EGFR, pan-ERG, FOLH1, MMP9, MUC1, NCOA2, KLK3 (PSA), SMAD4, SPINK1, SPARC, TFF3, TGFB1, and VEGFA. Corresponding original PRISM-SRM results and summary of the statistical analysis are provided in Tables S8 and S9, respectively. The PRISM-SRM assays were then refined, targeting the measurement of only these 16 proteins (see Section 4), testing their ability to discriminate between samples derived from tissue samples representing DM, BCR, and non-progression.

### 2.3. Determining Predictive Ability of Protein Biomarkers for Cancer Progression

For the final analyses, the entire cohort includes 338 patients (including the initial 105), of whom 53 (15.7%) experienced distant metastasis, 124 (36.7%) progressed to BCR, and 161 (47.6%) had no evidence of disease progression after a minimum of 10 years of follow-up time (Table 1). Median patient age at PCa diagnosis and post-RP follow-up times were 59.5 and 12.5 years, respectively. Median times from RP to BCR and metastasis were 1.7 and 6.7 years, respectively.

Several notable and expected differences were observed across the three event groups (Table 1), including poorer clinical features at time of PCa detection and poorer pathological features at time of RP for those who ultimately experienced disease progression (i.e., BCR and metastasis). This included higher pathologic T stage, higher grade group, and positive surgical margins at RP among those whose disease progressed. For those who developed metastasis, 91% had grade groups 4–5 at RP compared to 64% of those who developed BCR and only 39% of those in the nonevent group (*p* < 0.0001). No racial differences were noted across event status (*p* = 0.59), despite robust representation of African Americans.

Area under the receiver-operating characteristic curve (AUC) statistics are shown in Table 2 for each of the selected 16 protein markers for predicting metastasis (yes versus no) and BCR (yes versus no) events (box and whisker plots are shown in Figure S3) as well as for discriminating high versus low Gleason Group (GG) (i.e., 4–5 versus 1–3) [18]. Bonferroni correction for multiple comparisons (*p* = 0.05/16 = 0.0031) was used to ascertain statistical significance. Three proteins were statistically significant predictors across all 3 endpoints (i.e., metastasis, BCR, and GG) including FOLH1, SPARC, and TGFB1. In addition, decreases in tissue PSA levels was predictive of distant metastasis, and increases in CAMKK2, EGFR, and NCOA2 were also predictive of high GG.

**Figure 1:**
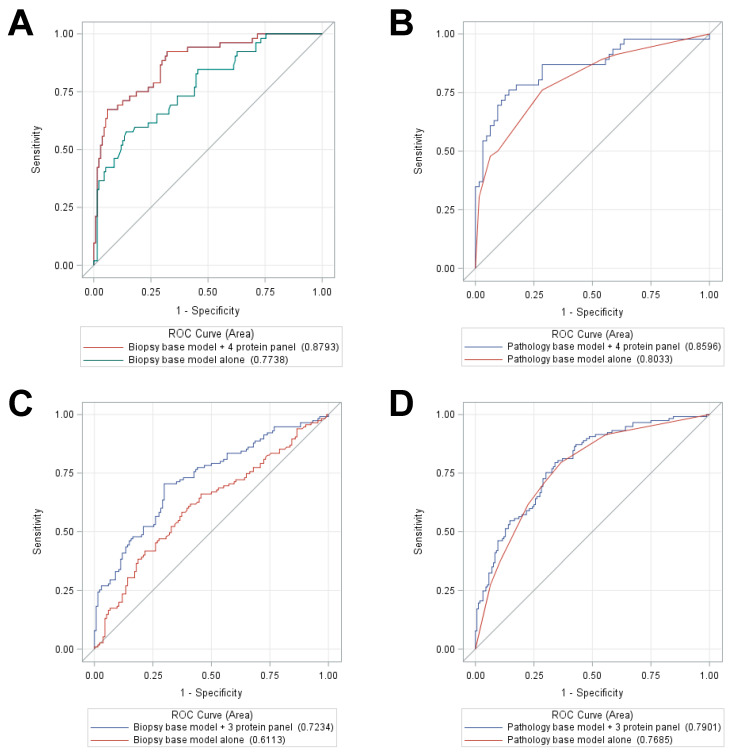
Receiver-operating characteristic (ROC) curves predicting DM or BCR using Standard of Care (SOC) base models and the protein panels versus SOC base models alone: ROC curve analyses for DM with comparison of AUC values for biopsy and pathology SOC base models alone versus in combination with the 4-protein marker panel are shown in (**A**) and (**B**), respectively. Similar analyses for BCR are shown in (**C**) and (**D**).

Using findings from Table 2, receiver-operating characteristic (ROC) curve analyses were examined for the primary study endpoints of disease progression, using only the proteins that achieved statistical significance for each study endpoint, including 4 protein markers for DM and 3 markers for BCR (Figure 1A–D). Serum PSA alone was also added to each figure for comparison. These marker panels were then combined with two sets of SOC variables—one consisting of clinical variables determined at prostate diagnostic biopsy (“biopsy base model”) including age, race, and National Comprehensive Cancer Network (NCCN)-risk strata and the second set consisting of pathology variables measured at time of RP (“pathology base model”) including pathological T stage, GG, and surgical margin status. The curves were examined for base models alone and base models plus the protein marker panels to determine whether improvement in AUC values was noted when protein panels were modeled concomitantly with base models. In Figure 1A,B, distant metastasis was examined as a function of protein panel including FOLH1, SPARC, TGFB1, and PSA; when added to the biopsy base model, the AUC increased to 0.88 from 0.77 (Figure 1A), while in the pathology base model, the AUC increased to 0.86 from 0.80 (Figure 1B). In Figure 1C,D, BCR was similarly examined as a function of a protein panel including FOLH1, SPARC, and TGFB1; when added to the biopsy base model, the AUC increased to 0.72 from 0.61 (Figure 1C), while in the pathology base model, the AUC increased to 0.79 from 0.77 (Figure 1D).

**Figure 2:**
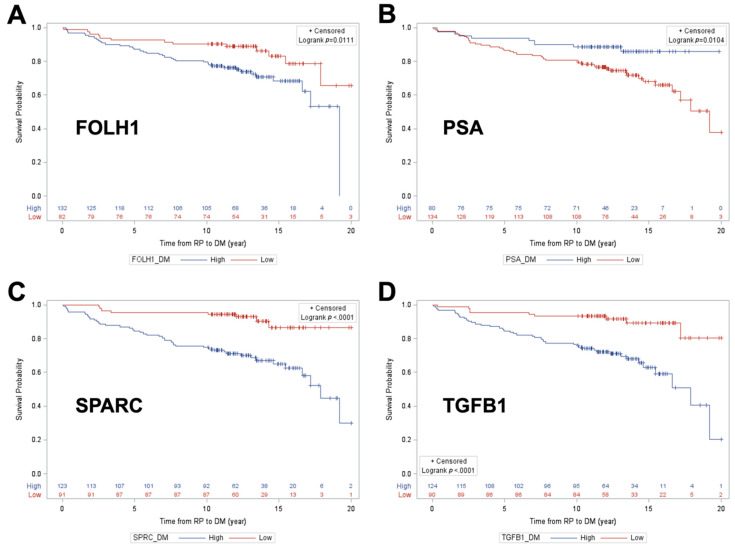
Kaplan–Meier DM-free survival curves across high versus low groups for FOLH1 (**A**), PSA (**B**), SPARC (**C**), and TGFB1 (**D**).

**Figure 3:**
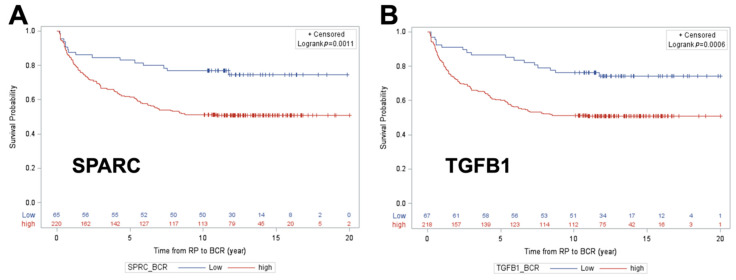
Kaplan–Meier BCR-free survival curves across high versus low groups for SPARC (**A**) and TGFB1 (**B**).

Optimal cut-points for each of these proteins were then identified (Table 3 and Table 4) [19]. Only markers with the highest sensitivity which could simultaneously achieve at least 70% negative predictive value (NPV) and 30% specificity were retained for subsequent analysis. For distant metastasis, this included all 4 protein markers from Table 2, while for BCR, this included only SPARC and TGFB1. Unadjusted Kaplan–Meier estimation curve analysis was then conducted to examined distant metastasis-free survival (DMFS) (Figure 2A–D) and BCR-free survival (Figure 3A,B), as a function of each protein marker, dichotomized at optimal cut-points identified in Table 3 and Table 4, specific to each study endpoint. All 4 protein markers were individually predictive of DMFS with the following directionalities of effect: higher FOLH1 (*p* = 0.011), SPARC (*p* = <0.0001), and TGFB1 (*p* < 0.0001) levels were predictive of poorer outcome, while lower PSA levels (*p* = 0.0104) were predictive of poorer DMFS outcome (Figure 2A–D). Significant predictors of BCR-free survival include higher levels of SPARC (*p* = 0.0011) and TGFB1 (*p* = 0.0006); both were predictive of poorer outcome (Figure 3A,B).

### 2.4. Training and Testing Set Analysis of a 5-Protein Classifier to Predict Distant Metastasis

In an effort to develop a proteomic classifier to predict DM, 214 patients (53 DM and 161 nonevents) were randomly split into training and testing cohorts (70% versus 30%) [20,21]. The training cohort consisted of 149 patients (33 DM and 116 nonevents), and the testing cohort consisted of 65 patients (20 DM and 45 nonevents) (Table S10). The comparison of distribution of clinical-pathological variables between training and testing cohorts are provided in Table S11. There was no significant difference in the distribution of clinical-pathological variables between the training and testing cohorts, except that the testing cohort had slightly shorter median follow up time than training cohort (*p* = 0.049). NCCN risk strata, pathological T stage, RP GG, and surgical margins status showed significant associations with distant metastasis in both the training and testing cohorts.

In the training cohort, univariable logistic regression analysis was used to select those proteins which significantly predicted DM. This included CAMKK2, FOLH1, PSA, SPARC, and TGFB1. Then, multivariable logistic regression modeling was performed using those 5 proteins (CAMKK2, FOLH1, PSA, SPARC, and TGFB1) to obtain parameter estimates to construct a 5-protein classifier for predicting DM, scaled from 0 to 100. Bootstrapped multivariable logistic regression (1000 replicates) was used with 1000 replicates to produce 95% confidence intervals for the optimal threshold for the protein classifier in predicting distant metastasis. The optimal threshold was defined as a cut point which maximizes sensitivity, with at least a 90% NPV and at least a 35% specificity [22]. Finally, this protein classifier and its threshold were analyzed in the testing cohort. The protein classifier performance, in both the training and testing cohorts, is presented in Figure S4 and Table S12. AUCs of the 5-protein classifier for DM in both the training and testing cohorts were 0.84 and 0.87, respectively (Figure S4A). In the testing cohort, the protein classifier cut-point of 8.3 generated a 92% NPV and a 90% sensitivity, with a 53% specificity for predicting DM (Table S12).

Finally, multivariable Cox proportional hazard analysis was used to examine the 5-protein classifier in predicting DMFS, controlling for variables of the biopsy base model (Table 5) and pathology base model (Table 6). In both the biopsy and pathology base models, the 5-protein classifier was treated first as dichotomized at threshold value (≥8.3 vs. <8.3) and then as a continuous variable. For all 4 models, the proportional hazards assumption of each covariate was tested and met. In the biopsy base model, patients with a high versus low protein classifier value (≥8.3 vs. <8.3) had significantly worse DMFS (HR = 5.09, 95% CI: 1.11–23.4, *p* = 0.036). When modeled continuously, a one-unit increase in the protein classifier value was significantly predictive of DMFS, when adjusting for biopsy base model variables (HR = 1.03, 95% CI: 1.02–1.05), *p* < 0.001) and pathology base model variables (HR = 1.02, 95% CI: 1.01–1.05, *p* = 0.018). The Kaplan–Meier DMFS curves across dichotomized 5-protein classifier groups (high vs. low) in the testing cohort is shown in Figure S5.

Similarly, multivariable Cox proportional hazard modeling for the classifier to predict BCR by adding the 5-protein classifier to biopsy and pathology SOC in testing cohort, and the Kaplan–Meier BCR-free survival curves across dichotomized 5-protein classifier groups (high vs. low) in the testing cohort are shown in Tables S13 and S14 and Figure S6, respectively.

When the Cancer of the Prostate Risk Assessment (CAPRA) risk score variables [23] were substituted for base model variables, the strength and directionality of effect between the 5-protein classifier and DMFS were consistent. However, in the model in which the 5-protein classifier was treated dichotomously, the association between the classifier and DMFS did not achieve statistical significance, with wide confidence intervals, demonstrating imprecise HR estimates (Table S15). It is important to note that several patients were missing data needed for CAPRA score calculation, reducing the effective sample size as well as the number of metastatic events and affecting the ability to show an association. Independently, CAPRA risk score did not predict DMFS. The Memorial Sloan Kettering Cancer Center (MSKCC) nomogram variables [24] were also evaluated in substitution for our base model variables. However, among the MSKCC required pathological predictors, seminal vesicle invasion demonstrated 100% correlation with the 5-protein classifier and, therefore, could not be modeled simultaneously with it to predict study outcomes (Tables S16 and S17).

## 3. Discussion

Improved clinical management of prostate cancer is based not only on early detection of neoplastic lesions in the prostate but also very significantly on the early discrimination of indolent prostate cancer, which can be effectively managed by active surveillance from aggressive forms of prostate cancer which could rapidly progress to castration resistance and/or metastatic disease. This project focused on identifying robust biomarkers to predict patients at risk for future cancer progression, including biochemical recurrence and distant metastasis, to enhance existing biopsy or pathology factors used for risk stratification and to provide additional support for safe delay (i.e., active surveillance) of invasive treatment versus early, aggressive therapy.

The experimental approach in this study was designed to raise the threshold for biomarker selection in a series of filters, with each providing increased stringency. The starting panel of candidate biomarkers, chosen on the basis of curated knowledge, was initially filtered on the basis of differential expression at the mRNA level using NanoString. Targeted antibody-independent proteomic assays (PRISM-SRM assays) were then developed for the 42 differentially expressed genes and tested for the ability to detect the cognate proteins in FFPE-preserved RP specimens from a small patient cohort with long-term follow-up, documenting DM, BCR, and no progression of events. Of the 42 proteins which could be detected and quantified, 16 were identified as differentially abundant across study outcomes in a combined cohort of 338 patients and finally in a testing-and-training cohort set. Three candidate proteins were robustly associated with BCR, four were associated with DM, and six were associated with high GG; across these proteins, three proteins were common in all endpoints: FOLH1, SPARC, and TGFB1. FOLH1, folate hydrolase 1, is also known as Prostate-Specific Membrane Antigen (PSMA) and has been extensively studied as a potential biomarker of PCa [25,26]. FOLH1 is involved in folate uptake, thus indirectly affects DNA synthesis, and is known to be correlated with aggressive PCa. SPARC, also known as osteonectin, has been frequently observed as an upregulated protein in multiple cancers including PCa [27,28]. SPARC/osteonectin plays an important role in bone mineralization and may serve to facilitate the metastasis of PCa to bone. TGFB1, transforming growth factor beta1, is a ubiquitous cytokine with well-documented effects on immune function that have been associated with tumor-promoting stromal interactions [29,30]. Thus, the consistent observation of these three proteins across models serves to further support their importance in tumor biology.

The final study cohort of 338 patients is one of the largest tested for validation of such proteomic biomarkers, and the final training and testing analysis ensures a thorough examination of the performance of the developed classifier. Moreover, it is important to note that the biomarker measurements in all the case-control studies presented in this report were solely based on antibody-independent MS assays. Such assays are highly sensitive, are precise, and circumvent the necessity to develop a high-quality ELISA for each of the candidate markers [16,31,32,33,34]. Albeit, an important limitation that must be noted is the possibility of model overfitting, since a limited number of study endpoints were examined in this single study.

**Figure 4:**
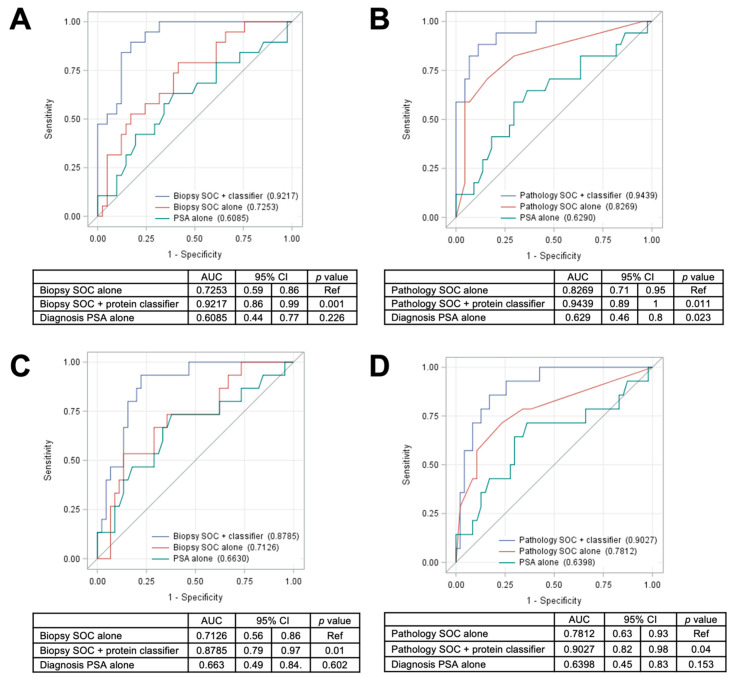
ROC curves predicting DM and BCR using SOC base models with versus without the protein classifier in the testing cohort: ROC curve analyses for DM with comparison of AUC values for biopsy and pathology SOC base models alone versus in combination with the 5-protein classifier are shown in (**A**) and (**B**), respectively. Similar analyses for BCR are shown in (**C**) and (**D**), respectively. The ROC curve analysis results using serum PSA alone were also shown as reference.

The current standard of care for PCa involves the initial use of biopsies and the biopsy base model to assess the risk of aggressive PCa in patients identified by PSA screening. Those patients exceeding a specific risk threshold are then referred for RP, and the pathological characteristics of the excised tumor are frequently used to guide further treatment. Improvements in the predictive power of the biopsy base model have the potential to significantly reduce the need for RP, and the tissue proteomic classifiers developed in this study significantly improved the performance of the biopsy base model for predicting either BCR or metastasis. Addition of the 5 protein classifier to the biopsy base model provided a significantly improved AUC of 0.92 (95% CI = 0.86–0.99, *p* = 0.001; versus 0.73 with the biopsy base model alone or 0.61 with PSA alone) for predicting DM (Figure 4A) as well as a significantly improved AUC of 0.88 (95% CI = 0.79–0.97, *p* = 0.01; versus 0.71 with the biopsy base model alone or 0.66 with PSA alone) for prediction of BCR (Figure 4C). While the tissue proteomic biomarkers did slightly improve the performance of the pathology base model for predicting both DM (Figure 4B) and BCR (Figure 4D), we postulate that the most valuable clinical application is likely to be in the analysis of prostate biopsy samples, prior to the acquisition of pathology metrics from RP. Although it remains to be demonstrated, prostate biopsy samples theoretically contain sufficient material for proteomic analysis of the panels developed in this study. If the predictive power of the proteomic classifier is also observed in biopsy samples, it may be possible to more accurately assess the risk of aggressive PCa from biopsy samples alone, potentially sparing RP in borderline risk scenarios. Although direct comparisons are complicated by differences in aspects such as study time period, cohort size, racial composition, specimen type, and methodologies, an initial comparison with the other existing commercial tests provides evidence of superior performance using the 5-protein classifier developed in this study (Table S18).

Prostate cancer molecular biomarkers, including prognostic ones, have been extensively reviewed [14,35,36,37,38,39,40]. The development of these biomarkers has created new opportunities for improving on current clinical practice but, at the same time, also poses challenges for the selection and incorporation of the most appropriate new assays into prostate cancer care. In addition to molecular biomarkers, prostate imaging (mpMRI) has recently emerged as a new tool to guide clinicians in managing prognostic evaluation of patients with localized prostate cancer [41,42,43]. An individualized risk-based decision making process needs to be established in the clinical practice, with contribution from both biomarkers and imaging [35,44,45]. Future research directions in support of this objective would include prospective studies of targeted protein marker expression in diagnostic biopsy specimens at the time when the option of active surveillance is still available [44,45,46].

## 4. Materials and Methods

### 4.1. Study Cohort

A retrospective cohort study was conducted using the Walter Reed National Military Medical Center (WRNMMC) prostate cancer Biospecimen Repository linked to the Center for Prostate Disease Research (CPDR) Multi-center National Database. In brief, specimens in the WRNMMC Biospecimen Repository were collected from PCa patients who underwent RP at WRNMMC and who provided informed consent to donate prostatectomy specimens to the repository and enrollment in the CPDR Multi-center National Database clinical data repository. The Multi-center National Database contains detailed demographic, clinical, treatment, pathologic, and outcomes information. Further details about these databases have been reported previously [47]. Both repositories and multi-center national database have Institutional Review Board (IRB) approval at the WRNMMC and the Uniformed Services University of the Health Sciences (USUHS), respectively. The IRB code for this study is Ref #908925 with an approval date of 18 June 2019 (at the Center for Prostate Disease Research).

### 4.2. Demographic, Clinical, and Treatment Variables

Variables were age at PCa diagnosis (years), self-reported race (African American, Caucasian American, and “Other”), PSA at PCa diagnosis (ng/mL), clinical T stage (T1-T2a, T2b-T2c, and T3a-T4), biopsy Gleason sum (≤6, 7, and 8–10), NCCN-defined risk strata (low, intermediate risk, and high risk), time from diagnosis to RP (months), and post-RP follow-up time (months).

### 4.3. RP Specimen Processing and Pathologic Variable Measurement

All RP specimens were processed by whole mount and sectioned at 2.2 mm intervals, as previously described [48,49]. Pathologic parameters were measured based on evaluation by central pathology review (by I.S.), including pathologic T stage (pT2 and pT3-pT4), grade group (GG1-5), and surgical margin status (negative and positive).

### 4.4. Dependent Study Outcomes

To ascertain whether targeted protein marker expression in FFPE tissues could be used to predict PCa progression, the study outcomes included BCR and DM after RP. A BCR event was defined in the following manner: a post-RP PSA level ≥0.2 ng/mL followed by a successive, confirmatory PSA level ≥ 0.2 or the initiation of salvage radiation or hormonal therapy after a rising PSA and excluding PSA values drawn within eight weeks of the RP. Presence of DM was ascertained by physician’s review of each patient’s complete imaging history, including bone scan, CT scan, MRI, and/or bone biopsy results. Subjects who had no evidence of BCR or metastasis at the end of study period with at least 10 years of post-RP follow-up were defined as “nonevents”.

### 4.5. Protein Digestion of FFPE Tissue Samples

The FFPE human prostate tissue samples were first deparaffinized by adding 500 µL of xylene (Sigma Aldrich, St. Louis, MO, USA) and by incubating for 5 min at room temperature with 300 rpm shaker speed. The solution was removed, and the xylene addition and incubation were repeated. After removing the solution for a second time, 500 µL of 190 proof ethanol (Decon Laboratories, King of Prussia, PA, USA) was added and incubated for 5 min at room temperature with 300 rpm shaker speed. The solution was removed. Finally, 500 µL of 80% ethanol was added and incubated for 5 min at room temperature with 300 rpm shaker speed. The solution was removed, and the samples were dried for 15 min in Speed-Vac (Thermo Fisher Scientific, Waltham, MA, USA).

Once dried, 50 µL of 2,2,2-Trifluoroethanol (TFE) (Sigma Aldrich) was added to the samples. Then, 50 µL of 600 mM Tris-HCl was added to the samples to give a final concentration of 50% TFE. The samples were homogenized with a Kontes^®^ Pellet Pestle^®^ (VWR, Radnor, PA, USA) for 30 s, keeping the samples cool on an ice block during homogenization and afterwards for 3 min. The samples were transferred to a 1.5-mL screw top tube before incubating with a Thermomixer (Eppendorf, Hamburg, Germany) at 99 °C for 90 min with 1000 rpm shaker speed. The samples were allowed to cool to room temperature. The protein concentrations of the samples were determined using bicinchoninic acid (BCA) assay (Thermo Fisher Scientific).

Proteins were reduced with 5 mM Dithiothreitol at 37 °C for 1 h and alkylated using 10 mM iodoacetamide at room temperature for 1 h in the dark. The samples were diluted with water and digested with sequencing grade modified trypsin (Promega Corporation, Madison, WI, USA) at a 1:50 trypsin:protein ratio. The samples were incubated at 37 °C for 4 h; then, a second 1:50 trypsin addition was made, and the samples were incubated overnight at 37 °C. The digestion was stopped by addition of 10% formic acid to have reach final concentration of 1% formic acid.

The samples were centrifuged at 14,000 rpm at 4 °C prior to the final solid-phase extraction (SPE) based desalting step using 50 mg, 1 mL C-18 SPE cartridges (Strata, Phenomenex, Torrance, CA, USA) and a manual vacuum manifold (Supelco, Sigma Aldrich). The cartridges were preconditioned using 3 mL of 100% methanol followed by 2 mL of 0.1% trifluoroacetic acid (TFA). The sample was loaded and slowly passed through the cartridge at a rate no faster than 1 mL per minute. The cartridge was then washed with 4 mL of 5% acetonitrile (ACN), 0.1% TFA, and 1 mL of 1% formic acid to remove any residual TFA. The desalted peptide sample was eluted into a 2.0-mL microcentrifuge tube using 1.5 mL of 80% can and 0.1% formic acid. The eluted sample was placed in the Speed-Vac and concentrated. The peptide concentration was determined using the BCA assay, and the final concentration was adjusted to 0.3 µg/µL. The sample was then frozen in liquid nitrogen and stored at −70 °C until needed for peptide spiking and SRM analysis.

### 4.6. PRISM-SRM Assay Configuration and Measurements

A total of 110 tryptic peptides for the 52 protein candidates were selected based on well-accepted criteria for targeted proteomics analysis [50]. Pure stable isotope-labeled heavy peptides (purity >97%) with C-terminal (U-13C6, 15N2) lysine or (U-13C6, 15N4) arginine were synthesized (AQUA QuantPro, ThermoFisher Scientific, Waltham, MA, USA) for PRISM-SRM assay development and measurements. The peptide list is provided in Table S2. The peptides were received at a concentration of 5 pmol/µL in 5% ACN. Equal volume of these 110 peptides were mixed together to create a heavy peptide mixture stock, and the final peptide concentration in the stock is 45 fmol/µL.

The transitions and collision energy of individual peptides were first optimized by direct infusion experiments on a TSQ Vantage triple quadrupole mass spectrometer (Thermo Fisher Scientific) and furthered evaluated by LC-SRM using a nanoACQUITY UPLC^®^ system (Waters Corporation, Milford, MA, USA) and a TSQ Vantage triple quadrupole mass spectrometer. Three best transitions with minimal interference and highest sensitivity were retained for each peptide in the final SRM assays. The list of best transitions, optimized collision energy, and LC elution times of each peptide are provided in Table S3.

Heavy peptides were spiked in digested and cleaned FFPE samples, and they were separated following the PRISM workflow using high pH reversed-phase capillary LC on a nanoACQUITY UPLC^®^ system as described previously [16]. Briefly, separations were performed using a capillary column packed in-house (3 μm Jupiter C18 bonded particles, 200 μm i.d. × 50 cm long) at a flow rate of 2.2 μL/min on binary pump systems, using 10 mM ammonium formate (pH 9) as mobile phase A and 10 mM ammonium formate in 90% ACN (pH 9) as mobile phase B. Forty-five µL of each sample (35 µg) were loaded onto the column and separated using a binary gradient of 5%–15% B in 15 min, 15%–25% B in 25 min, 25%–45% B in 25 min, and 45%–90% B in 38 min. The samples were separated into 96 fractions (1-min elution time per fraction), and the fractions were collected using a LEAP’s Collect PAL fraction collector (LEAP Technologies, Carrboro, NC, USA). Prior to peptide fraction collection, ~20 μL of water was added to each well in the plate to avoid peptide loss and to dilute the peptide fraction for LC-SRM analysis.

Configuration 1:110 peptides (52 proteins) in the first 105 samples. To facilitate the high-throughput PRISM-SRM analysis of 110 peptides in the first batch of 105 samples, the 96 fractions were concatenated into 24 fractions. These 24 fractions were analyzed individually on the second dimension LC-SRM using a nanoACQUITY UPLC^®^ system coupled to TSQ Vantage triple quadrupole mass spectrometer. Briefly, separations were performed using a ACQUITY UPLC M-Class Peptide BEH C18 Column, 300 Å, 1.7 µm, 100 µm × 100 mm (Waters Cooperation) at a flow rate of 0.4 μL/min and a temperature of 42 °C on binary pump systems, using 0.1% formic acid in water as mobile phase A and 0.1% formic acid in ACN as mobile phase B. Four µL of each sample was loaded onto the column at a flow rate of 0.5 µL/min for 10 min and separated using a binary gradient of 0.5–5% in 0.5 min, 5–20% B in 26.5 min, 20–25% B in 10 min, 25–38.5% B in 8 min, and 38.5–95% B in 1 min. The TSQ Vantage was operated with ion spray voltages of 2400 V and a capillary inlet temperature of 370 °C. Tube lens voltages were obtained from automatic tuning and calibration without further optimization. Both Q1 and Q3 were set at unit resolution of 0.7 FWHM, and Q2 gas pressure was 1.5 mTorr. A scan width of 0.002 m/z was used. Because of the large number of transitions to be scanned, we used a scheduled SRM method with RT window set to be 4 min and cycle time of 1 s.

Configuration 2: 16 peptides (16 proteins) in the remaining 233 samples. For the remaining 233 samples in the cohort, we reduced the number of protein candidates from 52 to 16. In order to achieve similar or even higher sensitivity with higher throughput, we selected only the target-containing fractions (roughly 16 fractions) during PRISM via online SRM monitoring of the heavy peptides, instead of concatenating into 24 fractions, for the second dimension LC-SRM analysis; we also used a faster LC gradient for the LC-SRM analysis of the PRISM fractions. Briefly, separations were performed at a flow rate of 0.4 μL/min and a temperature of 42 °C on binary pump systems, using 0.1% formic acid in water as mobile phase A and 0.1% formic acid in ACN as mobile phase B. Four µL of each sample was loaded onto the column at a flow rate of 0.5 µL/min for 10 min and separated using a binary gradient of 0.5–10% B in 0.5 min, 10–15% B in 1 min, 15–25% B in 6 min, 25–35% B in 3 min, and 35–95% B in 2 min. A nonscheduled SRM method with dwell time of 10 ms for each transition was used for analysis of the much smaller set of 16 peptides, while other MS conditions remained the same.

In order to evaluate the consistency between the first and second PRISM-SRM configurations, we used 3 individual samples from the first set of 105 and analyze them to quantify the final 16 peptides (of 16 proteins) using both configurations. The average measurement variations of 3 samples between two configurations for all the 16 peptides are between 2% and 29% with a median of 5%, which demonstrated the consistency in peptide quantification between two PRISM-SRM configurations.

### 4.7. Response Curves for the PRISM-SRM Assays

The sensitivity and linearity of the PRISM-SRM assay were determined by measuring heavy isotope-labeled peptide standards spiked into a sample pooled from all the remaining study samples to final concentrations of 0, 0.6, 3, 12, 60, 300, 1500, 3000, 6000, 12,000, 24,000 and 48,000 amol/µg. Each of the above samples were subjected to the same PRISM-SRM workflow as mentioned in configuration 2, with 3 injection replicates. The response curves of each peptide were generated using the heavy-over-light peak area ratios and the heavy peptides concentration as mentioned above. Signal-to-noise ratio (S/N) was calculated by the peak apex intensity over the highest background noise in a retention time region of ±15 s for the target peptides. The background noise levels were conservatively estimated by visually inspecting chromatographic peak regions. The lower limit of detection (LOD) and quantification (LOQ) were defined as the lowest concentration point of target proteins at which the S/N of surrogate peptides was at least 3 and 10, respectively. Additionally, LOQs also require a coefficient of variation (CV) of less than 20%. The final LOD and LOQ values of each assay were provided in Table S4. Note that, given the significant interference for the heavy peptide transitions of TGFB1 peptide GGEIEGFR (shown in Figure S2), the LOD and LOQ of GGEIEGFR cannot be accurately determined; however, we made sure that the S/N ratios of the endogenous (light) peptides are acceptable through manual inspection.

### 4.8. SRM Data Analysis

The entire cohort of FFPE samples was randomized and analyzed by PRISM-SRM at the Pacific Northwest National Laboratory (PNNL) in a blinded fashion (patient outcome data were restricted at CPDR during the entire analysis from the experimental design through statistical analysis). The raw data acquired on the TSQ Vantage triple quadrupole MS were imported into Skyline software (Version 20.1) [51] for visualization and quantification. The total peak area ratio between endogenous peptides and their heavy isotope labeled peptide standards was used for quantification. Peak detection and integration were based on two criteria: (1) the same retention time and (2) approximately the same relative peak intensity ratios across multiple transitions between endogenous peptide and heavy isotope labeled peptide standards. All data were manually inspected to ensure correct peak detection and accurate integration.

### 4.9. Endogenous Concentration Calculation

The final endogenous peptide concentrations (amol/µg) for all the samples were calculated using the response curves. The steps we took to calculate the final concentration of peptides in the study samples are provided below.

Step 1. Fit the calibration curve using linear regression (Y = *Slope* × X + *Intercept*), where X is the heavy peptide concentration in amol/µg and Y is the heavy over light peak area ratio (H/L). The final *slope* and *intercept* values are provided in Table S4.

Step 2. Calculate light peptide concentration of each peptide in the matrix (*C_light in response curve_*) using the response curve obtained above and data at three heavy peptide spike-in levels (300, 1500, and 3000 amol/µg) and obtain the average of calculated light peptide concentrations (amol/µg).

Step 3. Calculate the final endogenous peptide concentration in the study samples (*C_endogenous in sample_*) using the *slope*, *intercept*, and *C_light in response curve_* of the response curves. Equation (1) is as follows:

(1) Cendogenous in sample=(L/H Ratioin sampleClight in response curveCheavy in sample−Intercept )/Slope

where *L/H Ratio_in sample_* is the light over heavy peptide peak area ratio (Supplementary Materials Table S5) obtained directly from PRISM-SRM measurements and *C_heavy in sample_* is the heavy peptide concentration spiked in the study samples (amol/µg) (Supplementary Materials Table S6). The final endogenous concentrations of the peptides in all the study samples are provided in Table S7.

### 4.10. Initial Evaluation of Performance of Protein Biomarker Panels

All statistical analysis was performed using SAS version 9.4 (Cary, NC, USA), and statistical significance was set at *p* < 0.05 (except for univariable analysis of individual protein markers, described below, that underwent Bonferroni adjustment).

Analysis of variance (ANOVA) and Kruskal–Wallis testing was used to examine differences in the distribution of continuous variables (i.e., age at diagnosis, time from diagnosis to RP, and total follow-up time) across event groups, while Chi-square or Fisher exact tests were used to evaluate the associations of categorical variables (e.g., self-reported patient race, PSA at diagnosis, clinical T stage, biopsy grade, NCCN risk stratum, pathological T stage, Grade Group (GG), and surgical margin status) across event groups.

AUC values were computed for each of the 16 proteins markers for the ability of the markers to discriminate event group comparisons, including distant metastasis vs. nonevents, BCR versus nonevents, and high versus low GG (3–5 vs. 1–2). Only markers that achieved an AUC value ≥0.60 were retained in subsequent models, per event group comparison. For predicting distant metastasis, among those markers with an AUC ≥ 0.60, an optimal cutpoint was derived for maximizing sensitivity while satisfying the following critical thresholds: Negative Predictive Value (NPV) > 80% and Specificity > 40%. The 95% confidence intervals for each AUC value were constructed using nonparametric, bootstrapping with 1000 replicates. Using the Bonferroni method, the type I error threshold for determining statistical significance was adjusted for evaluating 16 protein markers as follows: *p* = 0.05/16 = 0.003125.

Unadjusted Kaplan–Meier survival curve analysis with log-rank testing was used to examine distant metastasis-free survival as a function of each individual protein classifier cut point that achieved the NPV/Spec requirements, including FOLH1, PSA, SPARC, and TGFB1 for DM. In KM analysis for BCR-free survival, the same approach was used for two protein classifier cut points: SPARC and TGFB1.

ROC curves were then examined to predict distant metastasis and BCR, using SOC variables, alone and then in combination with protein panels constructed by using principal components analysis to identify panel candidates, defined as those markers that achieved a principal components Eigenvalue > 1 per study endpoint (BCR and DM). For DM, four proteins (FOLH1, PSA, SPARC, and TGFB1) were selected from univariable analysis to form a protein panel; for BCR, three proteins (FOLH1, SPARC, and TGFB1) were selected. Two types of SOC models were examined: (i) a base model with biopsy features (i.e., age at PCa diagnosis, patient race, and NCCN risk stratum) and (ii) a base model with pathology variables (i.e., age at PCa diagnosis, patient race, pathological T stage, GG, and surgical margin status). The comparison between the AUCs statistic for the base models versus base models in combination with protein panels, for predicting of each study endpoint (DM and BCR) was performed using maximum likelihood ratio tests.

Finally, for multivariable logistic regression analysis, ROC curves were generated to evaluate prediction accuracy based on AUC statistics of each set of protein marker “panels” for each outcome: metastasis, BCR, and high GG. Four multivariable Cox proportional hazards (PH), models were constructed to examine distant metastasis-free survival and BCR-free survival, each as a function of the protein markers specific to the study endpoint, adjusting first for the biopsy “base” SOC variables, followed by adjustment for the pathologic “base” SOC variables.

### 4.11. Protein Classifier Development and Evaluation

Among the 338 patients, 53 developed DM after RP, 124 developed BCR but did not metastasize, and 161 are nonevents (did not develop BCR or metastasis after at least 10 years of follow-up post-RP). Two hundred and fourteen patients (53 DM and 161 nonevents) were used to develop a biomarker panel classifier to predict DM. This 214-patient cohort was randomly split into training and testing data sets (70% vs. 30%): a training cohort of 149 patients (33 DM and 116 nonevents) and a testing cohort of 65 patients (20 DM and 45 nonevents).

Biomarker selection, classifier construction, and optimal cutoff development in the training cohort were performed as followed: (1) Biomarker selection: Univariable logistic regression analysis was used to select those biomarkers which are significantly predicting DM status (*p* < 0.05 and AUC > 0.65). They are CAMKK2, FOLH1, PSA, SPRC, and TGFB1; (2) protein classifier construction: Fitted multivariable logistic regression model including the 5-protein panel to get parameter estimates for 5-protein panel classifier construction in predicting DM, scaled from 0 to 100; (3) classifier threshold: bootstrapped multivariable logistic regression (with 1000 replicates) was used to search for optimal cutoff of protein classifier in predicting DM, and the optimal threshold was defined as a cut point which maximizes sensitivity, with at least 90% NPV and at least 35% specificity.

Testing of the protein classifier and its threshold in the testing cohort was performed as followed: (1) Protein classifier testing: those parameter estimates which were generated in training cohort were applied to the testing cohort to construct a protein classifier. First, we tested 5-protein panel classifier’s prediction accuracy in predicting DM using logistic regression and ROC curve. Second, we evaluated the effects of adding the 5-protein classifier to biopsy SOC model (age, race, and NCCN-risk strata) and pathology SOC models (pathological T stage, GG, and surgical margin) on prediction accuracy based on PSA, using multivariable logistic regression analysis, ROC curve, and Mantel–Haenszel Chi-square tests. (2) Classifier threshold testing: The threshold for the 5-protein panel classifier in predicting DM-free survival was tested using univariable Kaplan–Meier curve and log-rank test. The classifier was tested alone, and when added to both the biopsy SOC model and the pathology SOC models, using multivariable-Cox proportional hazard analysis, the proportional hazard assumption of each covariate was checked and met.

The same method was used to evaluate the performance of the classifier for predicting BCR in the training and testing cohorts.

### 4.12. Data Availability

The data generated or analyzed during this study are included in the published article and its supplementary files. The PRISM-SRM results in Skyline format can be viewed and downloaded from Panorama (https://panoramaweb.org/Prostate_Biomarker_PNNL_2019.url).

## 5. Conclusions

In this study, we have used highly sensitive, antibody-independent, and multiplexed PRISM-SRM assays for verifying 52 genomic biomarker candidates at the protein level in primary tumors from a large prostate cancer patient cohort. A 5-protein classifier (FOLH1, PSA, TGFB1, SPARC, and CAMKK2) was developed for separating DM, BCR, and non-progression patients using training and testing cohorts. Potential applications of this novel protein classifier include early detection of aggressive prostate cancers and improved risk stratification for patients who could be managed through active surveillance.

The significant increase in performance over the use of the standard of care biopsy model, suggests that the application of the these protein biomarkers at the time of diagnostic biopsy may be sufficient to triage men to active surveillance without the need for a radical prostatectomy or extensive follow-up biopsies, substantially improving their quality of life. The ability to improve over the current standard of care pathology model provides further evidence that this test may help in primary treatment decision.

## Figures and Tables

**Table 1:** Descriptive characteristics of study cohort stratified by event status (n = 338).

Variable	Total	Nonevent	BCR ^*^	Metastasis	*p* Value
N	338	161	124	53	
Age at diagnosis (years)					
Mean (SD)	59.5 (7.7)	59.0 (8.1)	59.2 (7.7)	61.7 (5.9)	0.0897
Time from diagnosis to RP ^*^ (months)					
Median (range)	2.3 (0.2–21)	2.2 (0.2–21)	2.5 (0.2–9)	2.0 (0.7–10)	0.4689
Race					
AA ^*^	120 (35.6)	55 (34.2)	48 (39.0)	17 (32.1)	
CA ^*^ and Other	217 (64.4)	106 (65.8)	75 (61.0)	36 (67.9)	0.5882
PSA ^*^ at diagnosis (ng/mL)					
<10	262 (78.0)	133 (83.6)	90 (72.6)	39 (73.6)	
10–20	59 (17.6)	25 (15.7)	25 (20.2)	9 (17.0)	
>20	15 (4.5)	1 (0.6)	9 (7.3)	5 (9.4)	0.0062
Clinical T stage					
T1-T2a	274 (82.0)	134 (85.4)	107 (86.3)	33 (62.3)	
T2b-T2c	52 (15.6)	22 (14.0)	15 (12.1)	15 (28.3)	
T3a-T4	8 (2.4)	1 (0.6)	2 (1.6)	5 (9.4)	0.0005
Biopsy grade					
6 or less	182 (58.3)	100 (70.9)	68 (57.1)	14 (26.9)	
7	95 (30.4)	35 (24.8)	41 (34.4)	19 (36.5)	
8–10	35 (11.2)	6 (4.3)	10 (8.4)	19 (36.5)	<0.0001
NCCN ^*^ risk					
Low	125 (40.6)	69 (50.7)	46 (38.3)	10 (19.2)	
Intermediate	134 (43.5)	59 (43.4)	55 (45.8)	20 (38.5)	
High	49 (15.9)	8 (5.9)	19 (15.8)	22 (42.3)	<0.0001
Pathological T stage					
pT2	174 (52.6)	119 (74.4)	46 (37.4)	9 (18.8)	
pT3-4	157 (47.4)	41 (25.6)	77 (62.6)	39 (81.2)	<0.0001
GG ^*^					
GG1	31 (9.3)	18 (11.2)	13 (10.6)	0	
GG2	105 (31.6)	77 (48.1)	27 (22.0)	1 (2.0)	
GG3	6 (1.8)	2 (1.2)	4 (3.2)	0	
GG4	124 (37.4)	54 (33.8)	49 (39.8)	21 (42.9)	
GG5	66 (19.9)	9 (5.6)	30 (24.4)	27 (55.1)	<0.0001
Surgical margin					
Negative	209 (63.7)	126 (79.2)	62 (51.2)	21 (43.8)	
Positive	119 (36.3)	33 (20.8)	59 (48.8)	27 (56.2)	<0.0001
Post-RP Follow-up (months)					
Median (range)	150 (18–253)	156 (121–252)	129 (18–229)	124 (24–253)	<0.0001

^*^ BCR: biochemical recurrence; RP: radical prostatectomy; AA: African American; CA: Caucasian American; PSA: prostate-specific antigen; NCCN: National Comprehensive Cancer Network; GG: Gleason Group.

**Table 2:** Individual area under the receiver-operating characteristic curve (AUC) and *p* values of 16 proteins to predict distant metastasis (DM), biochemical recurrence (BCR), or high Grade Group (GG): The significant *p* values (<0.003) are shown in bold font.

	DM vs. Nonevent		BCR vs. Nonevent		GG (3–5 vs. 1–2)	
Protein	AUC	*p* Value	AUC	*p* Value	AUC	*p* Value
ANXA2	0.535	0.741	0.538	0.341	0.499	0.692
CAMKK2	0.591	0.051	0.604	0.009	0.667	**<0.001**
CCND1	0.532	0.166	0.624	0.037	0.592	0.034
EGFR	0.628	0.012	0.578	0.035	0.653	**<0.001**
ERG	0.543	0.668	0.546	0.830	0.482	0.708
FOLH1	0.653	**0.001**	0.627	**<0.001**	0.657	**<0.001**
MMP9	0.562	0.518	0.511	0.770	0.554	0.643
MUC1	0.570	0.461	0.474	0.603	0.506	0.200
NCOA2	0.637	0.095	0.613	0.225	0.670	**0.001**
PSA	0.730	**0.001**	0.529	0.955	0.608	0.005
SMAD4	0.511	0.622	0.526	0.092	0.521	0.383
SPINK1	0.486	0.207	0.548	0.535	0.547	0.470
SPARC	0.800	**<0.001**	0.695	**<0.001**	0.715	**<0.001**
TFF3	0.541	0.174	0.472	0.578	0.492	0.751
TGFB1	0.788	**<0.001**	0.649	**<0.001**	0.705	**<0.001**
VEGFA	0.528	0.168	0.601	0.040	0.573	0.009

**Table 3:** Cut-point identification for distant metastasis (DM) by protein marker.

Protein	Cut-Point ^*^	95% CI ^**^	Sensitivity	Specificity	PPV ^***^	NPV
FOLH1	−0.54	−0.55, −0.53	0.731	0.419	0.325	0.803
PSA	−0.12	−0.15, −0.08	0.827	0.412	0.350	0.862
SPARC	−0.53	−0.55, −0.52	0.865	0.522	0.409	0.910
TGFB1	−0.50	−0.52, −0.48	0.846	0.493	0.389	0.893

^*^ Optimal cutoff was chosen a point value with the highest sensitivity among the cut points which satisfy at least 80% negative predictive value (NPV) and 40% specificity. ^**^ Boostrapping method was used with 1000 replicates to produce 95% confidence intervals. ^***^ PPV: positive predictive value.

**Table 4:** Cut-point identification for biochemical recurrence (BCR) by protein marker.

Protein	Cut-Point ^*^	95% CI ^**^	Sensitivity	Specificity	PPV ^***^	NPV
SPARC	−0.74	−0.75, −0.72	0.874	0.301	0.523	0.732
TGFB1	−0.71	−0.73, −0.69	0.866	0.309	0.523	0.724

^*^ Optimal cutoff was chosen a point value with the highest sensitivity among the cut points which satisfy at least 70% NPV and 30% specificity. ^**^ Boostrapping method was used with 1000 replicates to produce 95% confidence intervals. ^***^ PPV: positive predictive value.

**Table 5:** Multivariable Cox proportional hazard model predicting DM as a function of a 5-protein classifier to complement biopsy SOC variables in study testing cohort.

Variable	Model 1 ^*^			Model 2 ^**^		
	HR	95% CI	*p* Value	HR	95% CI	*p* Value
Age at diagnosis	1.00	0.93–1.07	0.898	1.03	0.96–1.11	0.407
Race (AA vs. CA)	0.94	0.33–2.74	0.916	1.59	0.54–4.64	0.396
Risk (intermediate vs. low)	2.31	0.69–7.76	0.176	1.49	0.41–5.47	0.545
Risk (high vs. low)	4.68	1.14–19.22	0.032	2.29	0.52–10.16	0.274
5-protein classifier (high vs. low)	5.09	1.11–23.38	0.036	1.03	1.02–1.05	<0.001

^*^ Model 1: Classifier was modeled dichotomously, using median split 8.3. ^**^ Model 2: Classifier was modeled as continuous.

**Table 6:** Multivariable Cox proportional hazard model predicting DM as a function of a 5-protein classifier to complement pathology SOC variables in study testing cohort.

Variable	Model 1 ^*^			Model 2 ^**^		
	HR	95% CI	*p* Value	HR	95% CI	*p* Value
Pathology T (pT3 vs. pT2)	2.54	0.78–8.27	0.122	1.94	0.52–7.15	0.321
GG (GG5 vs. GG1-4)	3.42	1.17–10.03	0.025	2.04	0.52–8.04	0.309
Surgical margin (Pos vs. neg)	1.31	0.47–3.68	0.603	1.23	0.42–3.57	0.705
5-protein classifier (high vs. low)	3.71	0.82–16.88	0.089	1.02	1.01–1.05	0.018

^*^ Classifier was modeled dichotomously, using median split 8.3. ^**^ Model 2: Classifier was modeled as continuous.

## Supplementary Materials

The following are available online at https://www.mdpi.com/2072-6694/12/5/1268/s1, Figure S1: Response curves of the PRISM-SRM assays of the 16 protein candidates (A–P), Figure S2: Interference in SRM detection of the heavy isotope-labeled internal standard for TGFB1 (GGEIEGFR), Figure S3: Box and whisker plots of 16 candidate protein marker expression levels across the event groups: DM, BCR, and nonevents with a minimum 10-year follow-up following RP, Figure S4: Performance of 5-protein classifier in predicting DM among training and testing cohorts, Figure S5: Kaplan–Meier DM-free survival curves across dichotomized 5-protein classifier groups (high vs. low) in testing cohort, Figure S6: Kaplan–Meier BCR-free survival curves across dichotomized 5-protein classifier groups (high vs. low) in testing cohort, Table S1: Probe sets designed for PCa prognostic marker discovery using NanoString technology (N = 151), Table S2: The 52 protein candidates and corresponding proteotypic peptides analyzed using LC-SRM and PRISM-SRM, Table S3: Details of the PRISM-SRM assay conditions, Table S4: The response curve results of the PRISM-SRM assays for the 16 protein candidates, Table S5: Light over heavy peptide peak area ratios of the 16 protein candidates in all the samples, Table S6: Heavy peptide concentration (amol/µg) spiked in the samples for PRISM-SRM analysis, Table S7: Calculated endogenous concentration (amol/µg) of the 16 protein candidates in all the samples, Table S8: Light over heavy peptide peak area ratios of the 42 protein candidates detected in an initial set of 105 samples, Table S9: Kruskal–Wallis testing results for the PRISM-SRM analysis of an initial set of 105 samples, Table S10: Clinical variable distribution between training and testing cohorts for predicting DM, Table S11: Distributions of clinical-pathological variables between nonevent and DM groups among training and testing cohorts, Table S12: Performance of optimal cutoff of DM risk score, Table S13: Multivariable Cox proportional hazard model predicting BCR by adding 5-protein classifier to biopsy SOC in testing cohort, Table S14: Multivariable Cox proportional hazard model predicting BCR by adding 5-protein classifier to pathology SOC in testing cohort, Table S15: Multivariable Cox proportional hazard model predicting DM as a function of protein classifier with CAPRA risk variables in study testing cohort (N_total_ = 47; N_met events_ = 15), Table S16: Correlation table to justify importance of not co-modeling Seminal Vesicle Involvement (SVI) with classifier for Memorial Sloan Kettering Cancer Center (MSKCC) comparison to Center for Prostate Disease (CPDR)-Pacific Northwest National Laboratory (PNNL) base models, Table S17: Multivariable Cox proportional hazard model predicting DM as a function of protein classifier with MSKCC risk variables except SVI in study testing cohort (N_total_ = 58; N_met events_ = 17), Table S18: Comparison of performance of commercial prostate cancer tests.
