# Supplementary material for: Proteomic Tissue-Based Classifier for Early Prediction of Prostate Cancer Progression
Source: Cancers (Basel). 2020 May 17;12(5):1268. doi: 10.3390/cancers12051268 (PMC7281161; doi:10.3390/cancers12051268)
Supplement: Supplementary file 1 [file cancers-12-01268-s001.zip › cancers-797182 supplementary final.docx]

Supplementary Material: Proteomic Tissue-Based Classifier for Early Prediction of Prostate Cancer Progression

Yuqian Gao, Yi-Ting Wang, Yongmei Chen, Hui Wang, Denise Young, Tujin Shi, Yingjie Song, Athena A. Schepmoes, Claire Kuo, Thomas L. Fillmore, Wei-Jun Qian, Richard D. Smith, Sudhir Srivastava, Jacob Kagan, Albert Dobi, Isabell A. Sesterhenn, Inger L. Rosner, Gyorgy Petrovics, Karin D. Rodland, Shiv Srivastava, Jennifer Cullen and Tao Liu

**This file includes:** Figures S1–S6, Tables S1, S9–S16**.** Tables S2 to S8 are in a separate Excel file “Table S2-S8.xlsx”

| 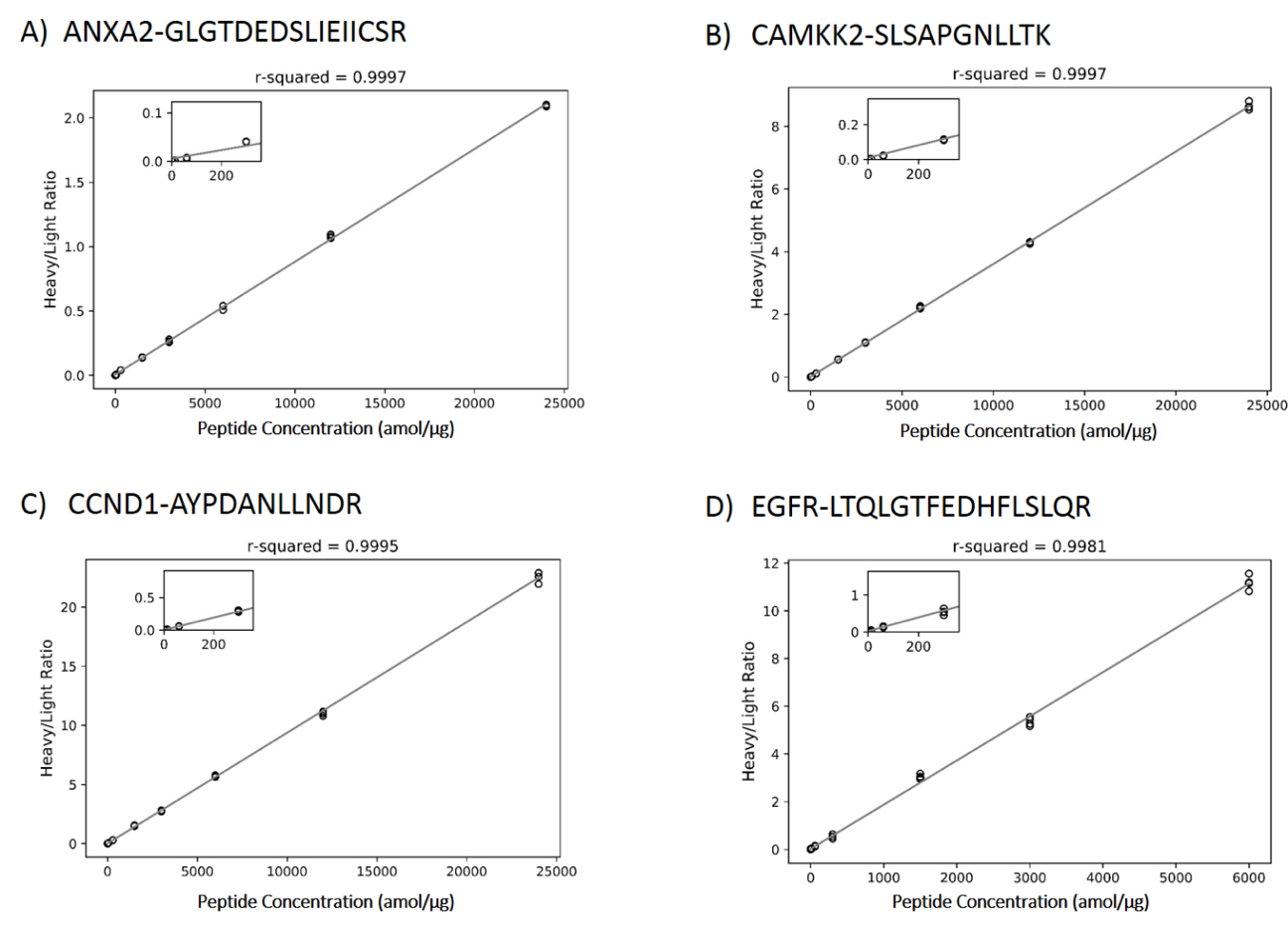 |
| --- |
| 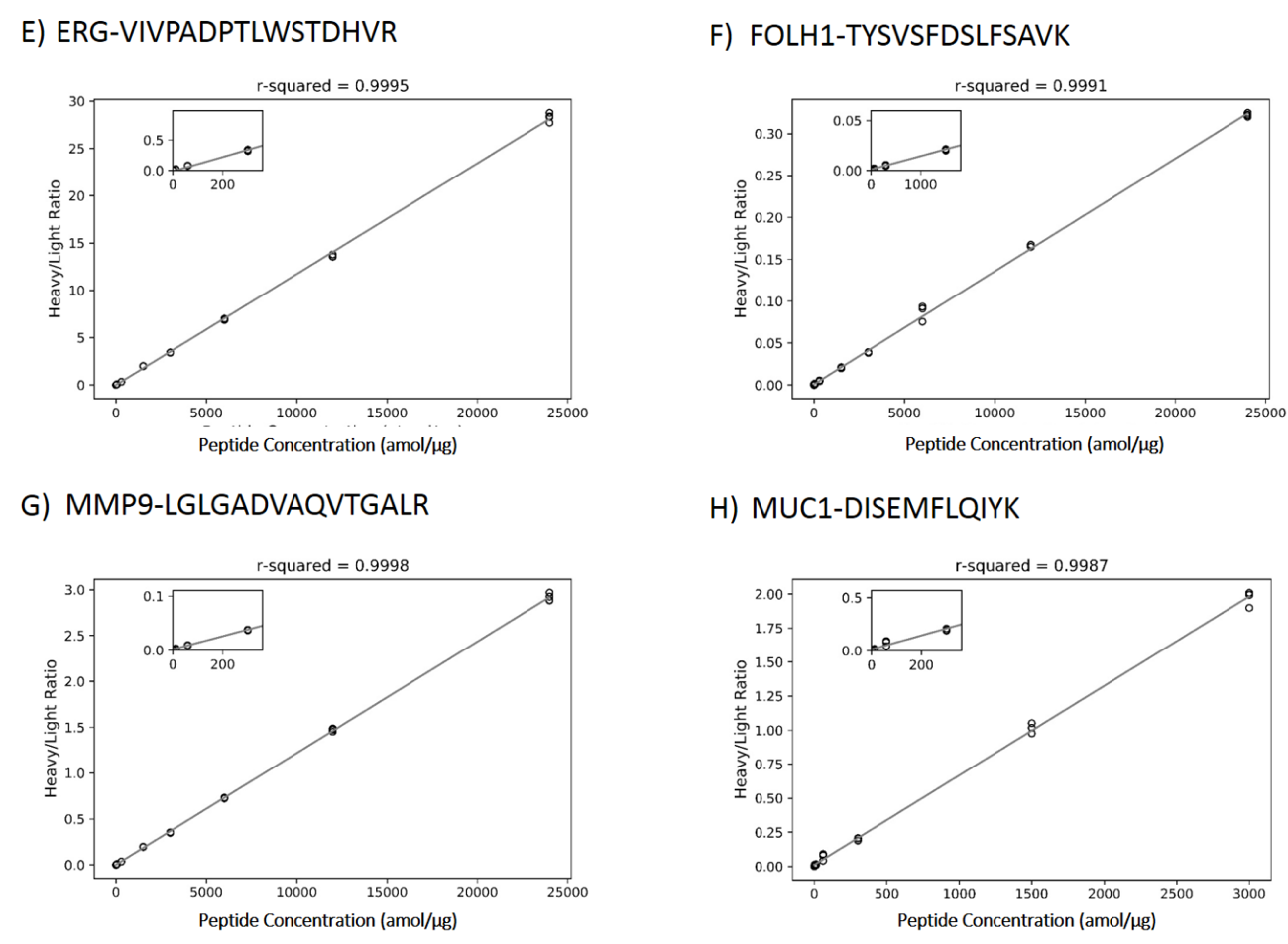 |
| 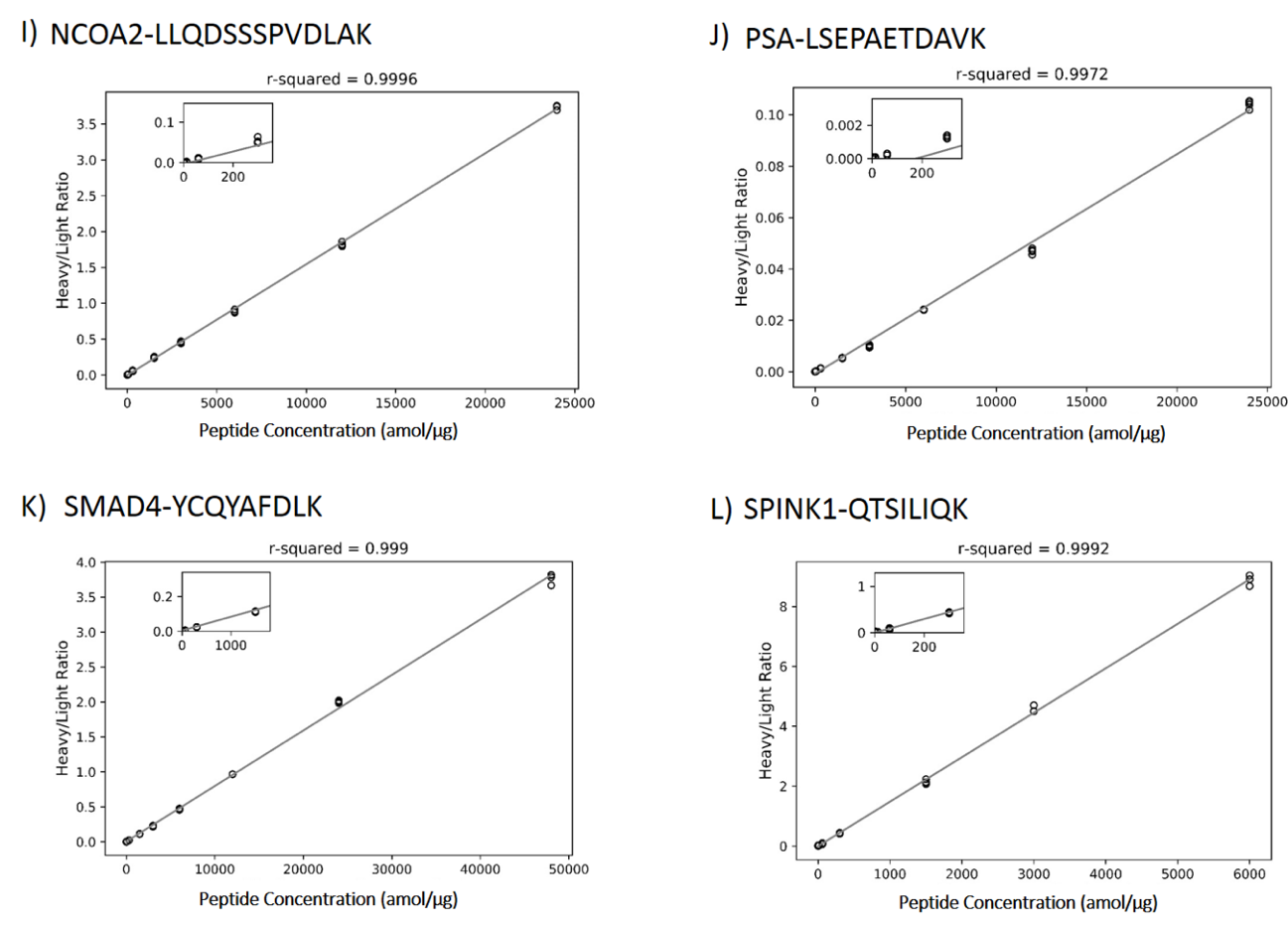 |
| 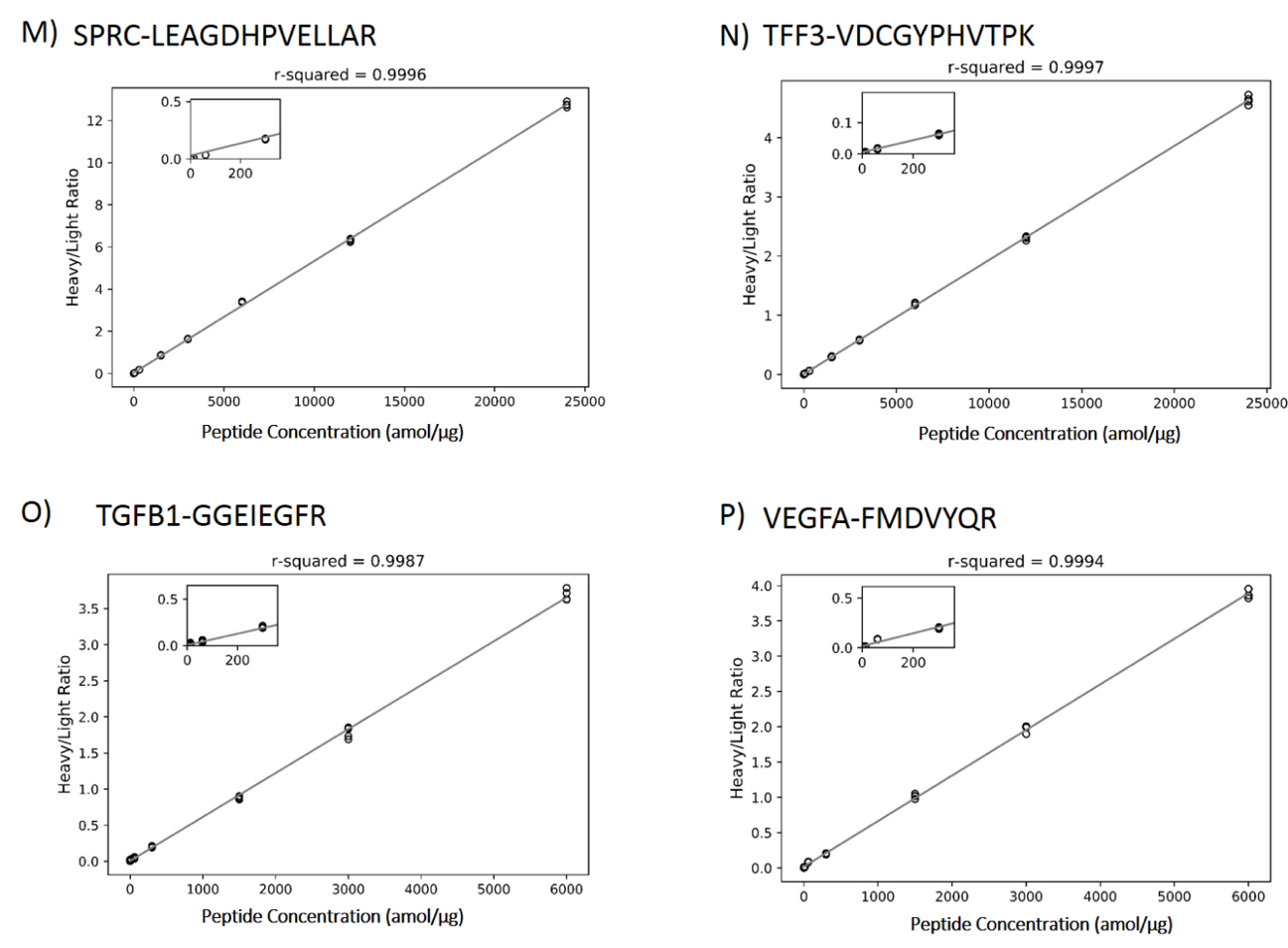 |

**Figure S1.** Response curves of the PRISM-SRM assays of the 16 protein candidates (**A**–**P**).


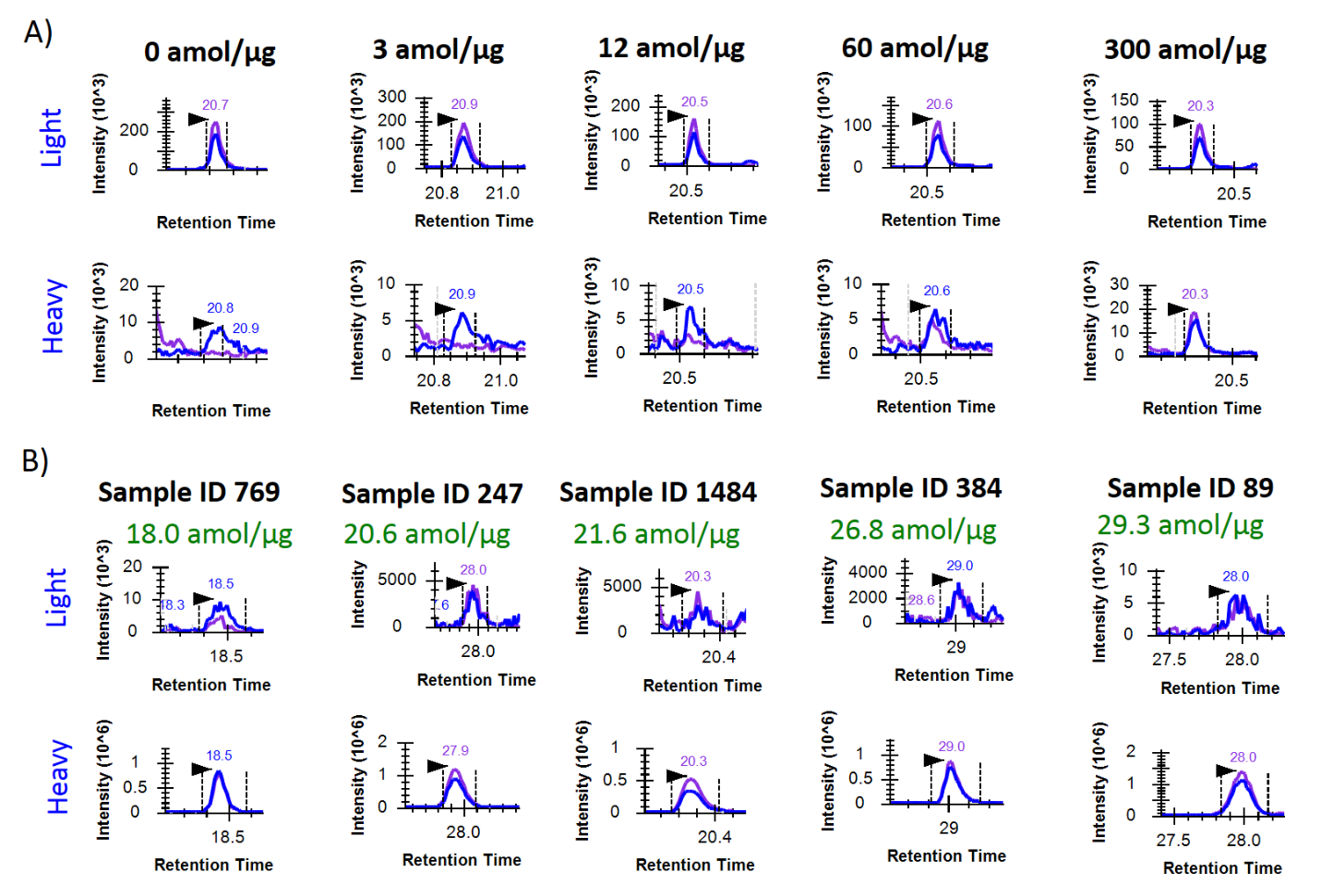


**Figure S2.** Interference in SRM detection of the heavy isotope-labeled internal standard for TGFB1 (GGEIEGFR). **A**) Example extracted ion chromatograms (XICs) of light and heavy peptides of TGFB1 peptide GGEIEGFR in various concentrations of the heavy peptide (0, 3, 12, 60, and 300 amol/ug) spiked in the matrix for the response curve analysis. **B**) Example XICs of light and heavy peptides of GGEIEGFR in five study samples that have the lowest levels of endogenous TGFB1 (i.e., light peptides). The blue trace indicates the transition from 432.7 to 621.3 (y5) while the purple trace indicates the transition from 432.7 to 379.2 (y3).


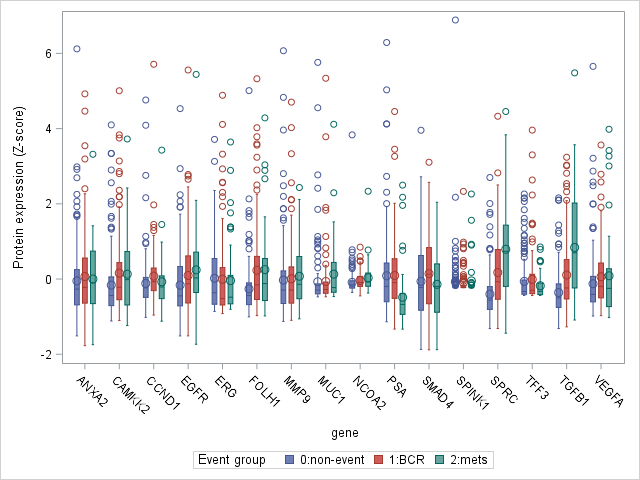


**Figure S3.** Box and whisker plots of 16 candidate protein marker expression levels across the event groups: distant metastasis (DM), biochemical recurrence (BCR), and non-events with a minimum 10-year follow-up following radical proctectomy (RP).

**Figure S4.** Performance of 5-protein classifier in predicting distant metastasis (DM) among training and testing cohorts. (**A**) Area under the curve (AUC) analysis in the training (blue) and testing (red) cohort. (**B**) Performance of the classifier on the training samples and testing samples. Negative predictive value (NPV) and specificity (SPC) are presented in terms of classifier risk score.


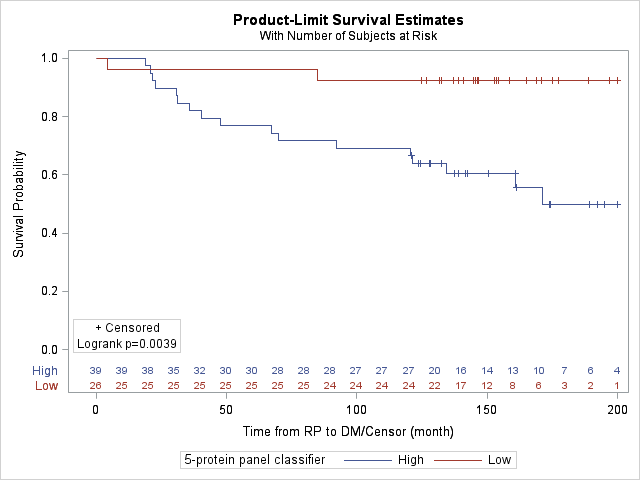


**Figure S5.** Kaplan-Meier distant metastasis (DM)-free survival curves across dichotomized 5-protein classifier groups (high vs. low) in testing cohort.


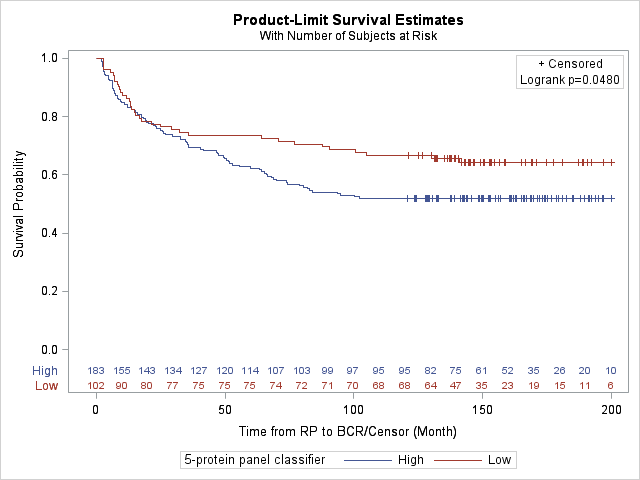


**Figure S6.** Kaplan-Meier biochemical recurrence (BCR)-free survival curves across dichotomized 5-protein classifier groups (high vs. low) in testing cohort.

**Table S1.** Probe sets designed for PCa prognostic marker discovery using NanoString technology (N = 151).

| **PCa** | **PCa Associated Genes** | | **PCa Prognosis** | **Cancer Gene** | **PCa** |
| --- | --- | --- | --- | --- | --- |
| **Gene Fusions** | **Up-regulated** | **Down-regulated** | **Associated Genes** | **Subset** | **Stromal/Epithelia Genes** |
| (ACSL3)3-(ETV1)6 | AMACR | AMD1 | AKT1 | AKT2 | ALCAM |
| (C15orf21)2-(ETV1)6 | BICD1 | EVA1 | ANXA2 | BRAF | KRT18 |
| (CANT1)1-(ETV4)5 | CACNA1D | GSTP1 | AR | CAV1 | KRT5 |
| (DDX5)2-ETV4)5 | CLDN8 | HOXB13 | AURKA | EGFR | POSTN |
| (FLJ35294)-(ETV1)5 | CRISP3 | KLK-2 | CAMK2N1 | FAS | Vimentin |
| (HERPUD1)1-E4 | EPC1 | KLK3/PSA | CCND1 | GATA1 | **ETS genes** |
| (HNRPA2B1)1-(ETV1)2 | EPC2 | LTF | CHD1 | HDAC1 | ESE3 |
| (KLK2)1-(ETV4)4 | ERG (pan) | MSMB | C-MYC | HIF1A | ETS 1 |
| (NDRG1)1-E4 | ERG1,2,3 | NEFH | COL1A1 | HRAS | ETS 2 |
| (SLC45A3)1-(ETV5)8 | ERG8 | NKX3.1 | COL3A1 | KRAS | FLI1 |
| (SLC45A3)1-E4 | ETV1 | ODC1 | CXCR4 | MMP-2 | PDEF |
| T1-(ETV4)2 | ETV4 | PAP | EZH2 | MMP-9 | **Housekeeping** |
| T1-E2 | ETV5 | PMEPA1 | FZD4 | NOTCH1 | ACTB |
| T1-E3 | GGT1 | Prostase (KLK-L1) | HSP27 | NRAS | B2M |
| T1-E4 | HOXC6 | PSCA | JAG1 | NUMA1 | CLTC |
| T1-E5 | HPGD | STAG1 | KLF4 | PDGFR | GAPDH |
| T2-E2 | MYO6 | TMPRSS2 | MAOA | PIK3CA | GUSB |
| T2-E4 | NPY |  | MUC-1 | RAF1 | HPRT1 |
| T2-E5 | PCA3 |  | MYCN | STAT1 | PGK1 |
| T3-(ETV5)2 | PCGEM1 |  | NCOA2 | STAT3 | RPL13A |
| T3-E4 | PLA2G7 |  | OCT4 | TP53 | RPL27 |
| T4-E4 | PSGR |  | PARP1 | VEGF | RPS13 |
| T4-E5 | PSGR2 |  | PTENP1 | VEGFR | TUBB |
| T5-E4 | PSMA/FOLH1 |  | PTEN | VEGFR1 |  |
| T5-E5 | SPARC |  | SMAD4 | WNT1 |  |
| AGTRAP-BRAF | TMEFF2 |  | SOX2 |  |  |
| SLC45A3-BRAF | TWIST1 |  | SPINK1 |  |  |
|  |  |  | SPP1 |  |  |
|  |  |  | SPRY1 |  |  |
|  |  |  | SPRY2 |  |  |
|  |  |  | STAG2 |  |  |
|  |  |  | TFF3 |  |  |
|  |  |  | TOP2A |  |  |
|  |  |  | ZEB1 |  |  |

**Table S2** (separate Excel file “Table S2-S8.xlsx”): The 52 protein candidates and corresponding proteotypic peptides analyzed using LC-SRM and PRISM-SRM.

**Table S3** (separate Excel file “Table S2-S8.xlsx”): Details of the PRISM-SRM assay conditions.

**Table S4** (separate Excel file “Table S2-S8.xlsx”): The response curve results of the PRISM-SRM assays for the 16 protein candidates.

**Table S5** (separate Excel file “Table S2-S8.xlsx”): Light over heavy peptide peak area ratios of the 16 protein candidates in all the samples.

**Table S6** (separate Excel file “Table S2-S8.xlsx”): Heavy peptide concentration (amol/µg) spiked in the samples for PRISM-SRM analysis.

**Table S7** (separate Excel file “Table S2-S8.xlsx”): Calculated endogenous concentration (amol/µg) of the 16 protein candidates in all the samples.

**Table S8** (separate Excel file “Table S2-S8.xlsx”): Light over heavy peptide peak area ratios of the 42 protein candidates detected in an initial set of 105 samples.

**Table S9.** Kruskal-Wallis testing results for the PRISM-SRM analysis of an initial set of 105 samples (20 DM, 37 BCR and 48 non-progression). A total of 42 proteins were detected, however 6 proteins (CDN1A, EZH2, CDN1A, DCOR, MUC6, OR51E2, and PAFA) were excluded from the analysis due to large proportion of missing values. The 16 proteins candidates selected in the full evaluation are shown in bold font).

| **Gene.** | ***p* value** | **Gene** | ***p* value** | **Gene** | ***p* value** |
| --- | --- | --- | --- | --- | --- |
| **TGFB1** | **0.00001** | **MMP9** | **0.1918** | MMP2 | 0.3740 |
| **SPARC** | **0.00004** | **TFF3** | **0.2260** | AR | 0.4365 |
| **CCND1** | **0.00796** | **ANXA2** | **0.2308** | HPN | 0.4541 |
| **PSA** | **0.00813** | **SPINK1** | **0.2512** | KLK11 | 0.5273 |
| **FOLH1** | **0.0163** | FGFR1 | 0.2543 | KLK2 | 0.5638 |
| **NCOA2** | **0.0237** | AMACR | 0.2607 | STAT3 | 0.6229 |
| **VEGFA** | **0.0546** | CRISP3 | 0.2767 | TPM2 | 0.7665 |
| **EGFR** | **0.0650** | BRAF | 0.2992 | PGFRB | 0.8062 |
| **SMAD4** | **0.0715** | TMPRSS2 | 0.3280 | NPY | 0.8362 |
| **MUC1** | **0.1463** | HSPB1 | 0.3391 | RAF1 | 0.8432 |
| **CAMKK2** | **0.1574** | AKT1 | 0.3543 | MYO6 | 0.8471 |
| **ERG_pan** | **0.1701** | SERPINI1 | 0.3700 | PIK3CA | 0.8620 |

**Table S10.** Clinical variable distribution between training and testing cohorts for predicting DM.

| **Clinical Variable** | **Training** | **Testing** | ***p* value** | **Pathological variable** | **Training** | **Testing** | ***p* value** |
| --- | --- | --- | --- | --- | --- | --- | --- |
| **N** | **149** | **65** |  | **N** | **149** | **65** |  |
| Age at diagnosis (yr) |  |  |  | Pathological T stage |  |  |  |
| Mean (SD) | 59.4 (7.8) | 60 (7.5) |  | pT2 | 91 (61.9) | 37 (60.7) |  |
| Median (range) | 60 (35-75) | 62 (42–75) | 0.419 | PT3 | 56 (38.1) | 24 (39.3) | 0.866 |
| Race |  |  |  | GG |  |  |  |
| AA | 53 (35.6) | 19 (29.2) |  | GG1-2 | 125 (84.5) | 48 (78.7) |  |
| CA&other | 96 (64.4) | 46 (70.8) | 0.367 | GG3-5 | 23 (15.5) | 13 (21.3) | 0.316 |
| PSA at diagnosis (ng/mL) |  |  |  | Surgical margin status |  |  |  |
| <10 | 116 (78.9) | 56 (86.2) |  | Negative | 102 (69.9) | 45 (73.8) |  |
| 10–19.99 | 26 (17.7) | 8 (12.3) |  | Positive | 44 (30.1) | 16 (26.2) | 0.573 |
| 20 or above | 5 (3.4) | 1 (1.5) | 0.200 | Upgrade |  |  |  |
| Clinical T stage |  |  |  | No | 43 (55.1) | 19 (52.8) |  |
| T2-T2a | 112 (76.7) | 55 (85.9) |  | Yes | 35 (44.9) | 17 (47.2) | 0.815 |
| T2b-c | 29 (19.9) | 8 (12.5) |  | Time from Dx to RP (mos) |  |  |  |
| T3a or above | 5 (3.4) | 1 (1.6) | 0.368 | Mean (SD) | 2.7 (2.1) | 3.1 (2.7) |  |
| Biopsy Gleason sum |  |  |  | Median (range) | 2.1 (0.2–21) | 2.2 (0.4–15) | 0.514 |
| 6 or less | 78 (59.1) | 36 (59.0) |  |  |  |  |  |
| 7 | 39 (29.5) | 15 (24.6) |  | Follow up after RP (mos) |  |  |  |
| 8–10 | 15 (11.4) | 10 (16.4) | 0.644 | Mean (SD) | 158.8 (40.0) | 148.9 (46.5) |  |
| NCCN risk |  |  |  | Median (range) | 157 (30–252) | 145 (24–253) | 0.049 |
| Low | 20 (15.6) | 10 (16.7) |  | Event group |  |  |  |
| Intermediate | 56 (43.8) | 23 (38.3) |  | Non-event | 116 (77.8) | 45 (69.2) |  |
| High | 52 (40.6) | 27 (45.0) | 0.766 | DM | 33 (22.2) | 20 (30.8) | 0.181 |

**Table S11.** Distributions of clinical-pathological variables between non-event and DM groups among training and testing cohorts.

| **Variable** | **Training** | | | **Testing** | | |
| --- | --- | --- | --- | --- | --- | --- |
|  | **Non-event** | **DM** | ***p* value** | **Non-event** | **DM** | ***p* value** |
| Age at diagnosis (yr) | 116 | 33 |  | 45 | 20 |  |
| Mean (SD) | 58.6 (8.1) | 62.3 (5.6) |  | 59.9 (8.0) | 60.5 (6.3) |  |
| Median (range) | 59 (35–75) | 62 (50–74) | 0.015 | 62 (42–75) | 61 (46–70) | 0.937 |
| Race |  |  |  |  |  |  |
| AA | 43 (37.1) | 10 (30.3) |  | 12 (26.7) | 7 (35.0) |  |
| CA&other | 73 (62.9) | 23 (69.7) | 0.475 | 33 (73.3) | 13 (65.0) | 0.498 |
| PSA at diagnosis (ng/mL) |  |  |  |  |  |  |
| <10 | 93 (81.6) | 23 (69.7) |  | 40 (88.9) | 16 (80.0) |  |
| 10–19.99 | 20 (17.5) | 6 (18.2) |  | 5 (11.1) | 3 (15.0) |  |
| 20 or above | 1 (0.9) | 4 (12.1) | 0.015 | 0 | 1 (5.0) | 0.277 |
| Clinical T stage |  |  |  |  |  |  |
| T2-T2a | 94 (83.2) | 18 (54.6) |  | 40 (90.9) | 15 (75.0) |  |
| T2b-c | 18 (15.9) | 11 (33.3) |  | 4 (9.1) | 4 (20.0) |  |
| T3a or above | 1 (0.9) | 4 (12.1) | <0.001 | 0 | 1 (5.0) | 0.138 |
| Biopsy Gleason sum |  |  |  |  |  |  |
| 6 or less | 69 (69.7) | 9 (27.3) |  | 31 (73.8) | 5 (26.3) |  |
| =7 | 28 (28.3) | 11 (33.3) |  | 7 (16.7) | 8 (42.1) |  |
| 8–10 | 2 (2.0) | 13 (39.4) | <0.001 | 4 (9.5) | 6 (31.6) | 0.001 |
| NCCN risk |  |  |  |  |  |  |
| Low | 46 (48.4) | 6 (18.2) |  | 23 (56.1) | 4 (21.0) |  |
| Intermediate | 45 (47.4) | 11 (33.3) |  | 14 (34.2) | 9 (47.4) |  |
| High | 4 (4.2) | 16 (48.5) | <0.001 | 4 (9.8) | 6 (31.6) | 0.005 |
| Pathological T stage |  |  |  |  |  |  |
| pT2 | 87 (75.0) | 4 (12.9) |  | 32 (72.7) | 5 (29.4) |  |
| PT3 | 29 (25.0) | 27 (87.0) | <0.001 | 12 (27.3) | 12 (70.6) | 0.002 |
| GG* |  |  |  |  |  |  |
| GG1-4 | 110 (94.8) | 15 (46.9) |  | 41 (93.2) | 7 (41.2) |  |
| GG5 | 6 (5.2) | 17 (53.1) | <0.001 | 3 (6.8) | 10 (58.8) | <0.001 |
| Surgical margin status |  |  |  |  |  |  |
| Negative | 91 (79.1) | 11 (35.5) |  | 35 (79.6) | 10 (58.8) |  |
| Positive | 24 (20.9) | 20 (64.5) | <0.001 | 9 (20.4) | 7 (41.2) | 0.115 |

Since there were only 1 patient developed DM among GG1-3 patients, GG1-4 patients were combined to one group for DM study.

**Table S12.** Performance of optimal cutoff of DM risk score.

| **5-protein Classifier Threshold** | **Training** | | | | **Testing** | | | |
| --- | --- | --- | --- | --- | --- | --- | --- | --- |
|  | **NPV (95% CI)** | **Sens (95% CI)** | **SPC (95% CI)** | **PPV (95% CI)** | **NPV** | **Sens** | **SPC** | **PPV** |
| 8.3 | 0.913 (0.911–0.915) | 0.879 (0.875–0.883) | 0.362 (0.350–0.374) | 0.282 (0.274–0.289) | 0.923 | 0.900 | 0.533 | 0.462 |

**Table S13.** Multivariable Cox proportional hazard model predicting BCR by adding 5-protein classifier to biopsy SOC in testing cohort.

| **Variable** | **Model 1 ^*^** | | | **Model 2 ^**^** | | |
| --- | --- | --- | --- | --- | --- | --- |
|  | **HR** | **95% CI** | ***p* value** | **HR** | **95% CI** | ***p* value** |
| Age at diagnosis | 1.00 | 0.98–1.02 | 0.998 | 1.00 | 0.97–1.02 | 0.723 |
| Race (AA vs. CA) | 1.18 | 0.81–1.71 | 0.399 | 1.20 | 0.82–1.74 | 0.346 |
| Risk (intermediate vs. low) | 1.25 | 0.84–1.86 | 0.271 | 1.09 | 0.73–1.64 | 0.667 |
| Risk (high vs. low) | 2.35 | 1.36–4.07 | 0.002 | 1.78 | 1.01–3.15 | 0.045 |
| 5-protein classifier (high vs. low) | 1.25 | 0.83–1.86 | 0.284 | 1.02 | 1.01–1.03 | <0.001 |

^*^ Model 1: Classifier was dichotomized at threshold of 8.3

^**^ Model 2: Classifier was continuous

**Table S14.** Multivariable Cox proportional hazard model predicting BCR by adding 5-protein classifier to pathology SOC in testing cohort.

| **Variable** | **Model 1 ^*^** | | | **Model 2 ^**^** | | |
| --- | --- | --- | --- | --- | --- | --- |
|  | **HR** | **95% CI** | ***p* value** | **HR** | **95% CI** | ***p* value** |
| Pathology T (pT3 vs. pT2) | 2.48 | 1.64–3.75 | <0.001 | 2.25 | 1.49–3.42 | <0.001 |
| GG (GG5 vs. GG1-4) | 2.21 | 1.43–3.42 | <0.001 | 1.93 | 1.23–3.04 | 0.004 |
| Surgical margin (Pos vs. neg) | 1.77 | 1.22–2.59 | <0.001 | 1.67 | 1.14–2.45 | 0.008 |
| 5-protein classifier (high vs. low) | 0.96 | 0.62–1.46 | 0.833 | 1.01 | 1.00–1.02 | 0.072 |

^*^ Model 1: Classifier was dichotomized at threshold of 8.3

^**^ Model 2: Classifier was continuous

**Table S15.** Multivariable Cox proportional hazard model predicting DM as a function of protein classifier with CAPRA risk variables in study testing cohort (N_total_ = 47; N_met events_ = 15).

| **Variable** | **Model 1 ^*^** | | | **Model 2 ^**^** | | |
| --- | --- | --- | --- | --- | --- | --- |
|  | **HR** | **95% CI** | ***p* value** | **HR** | **95% CI** | ***p* value** |
| Race (AA vs. CA) | 1.03 | 0.31–3.46 | 0.961 | 1.8 | 0.49–6.5 | 0.383 |
| CAPRA Risk (intermediate vs. low) | 2.7 | 0.7–10.1 | 0.154 | 1.4 | 0.3–6.6 | 0.698 |
| CAPRA Risk (high vs. low) | 4.9 | 0.96–25.4 | 0.056 | 2.5 | 0.4–14.9 | 0.313 |
| 5-protein classifier (high vs. low) | 3.7 | 0.8–16.9 | 0.09 | 1.03 | 1.01–1.05 | 0.008 |

**^*^** Model 1: classifier was dichotomized as high vs low, using median split 8.3

**^**^** Model 2: classifier was modeled as a continuous factor

Age is removed from the model because it is needed to calculate CAPRA score; 13 patients were lost from the model due to missing biopsy core data (n = 5) and missing Gleason score (n = 8), reducing the testing cohort from N = 60 to 47, and met events from n = 19 to 15.

**Table S16.** Correlation table to justify importance of not co-modeling Seminal Vesicle Involvement (SVI) with classifier for MSKCC comparison to CPDR-PNNL base models.

|  | **5-protein Classifier (dichotomous)** | | | | | **5-protein Classifier (continuous)** |
| --- | --- | --- | --- | --- | --- | --- |
|  | **High** | | **Low** | | ***p* value** | ***p* value** |
|  | **N** | **(%)** | **N** | **(%)** |  |  |
| PSA at diagnosis, median (min, max) | 5.9 (1, 17.5) | | 5.4 (1.7, 88.7) | | 0.516 |  |
| Age at RP, median (min, max) | 63.4 (44.7, 75.4) | | 60.3 (42.2, 69.1) | | 0.052 |  |
| Grade Group (GG) |  |  |  |  | 0.122 | 0.007 |
| GG1-GG4 | 24 | 70.6 | 23 | 88.5 |  |  |
| GG5 | 10 | 29.4 | 3 | 11.5 |  |  |
| Surgical margin status |  |  |  |  | 0.378 | 0.207 |
| Negative | 23 | 67.7 | 21 | 80.8 |  |  |
| Positive | 11 | 32.3 | 5 | 19.2 |  |  |
| Extracapsular Extension |  |  |  |  | 0.5889 | 0.146 |
| Negative | 20 | 57.1 | 16 | 66.7 |  |  |
| Positive | 15 | 42.9 | 8 | 33.3 |  |  |
| Seminal Vesicle Involvement |  |  |  |  | 0.017 | 0.0002 |
| Negative | 27 | 79.4 | 25 | 100 |  |  |
| Positive | 7 | 20.6 | 0 | 0 |  |  |

Note associations of SVI with classifier modeled as *categorical and continuous*.

**Table S17.** Multivariable Cox proportional hazard model predicting DM as a function of protein classifier with MSKCC risk variables except SVI in study testing cohort (N_total_ = 58; N_met events_ = 17).

| **Variable** | **Model 1 ^*^** | | | **Model 2 ^**^** | | |
| --- | --- | --- | --- | --- | --- | --- |
|  | **HR** | **95% CI** | ***p* value** | **HR** | **95% CI** | ***p* value** |
| PSA at diagnosis (ng/mL) | 1.1 | 1–1.1 | 0.014 | 1.1 | 1–1.2 | 0.019 |
| Age at RP (years) | 1.0 | 0.9–1.1 | 0.808 | 1.0 | 0.95–1.1 | 0.339 |
| GG (GG5 vs. GG1-4) | 3.2 | 1–9.8 | 0.046 | 1.1 | 0.3–4.7 | 0.852 |
| Surgical margin (+ vs. −) | 1.6 | 0.5–4.7 | 0.418 | 1.5 | 0.5–4.2 | 0.491 |
| Extracapsular Extension (+ vs. −) | 1.0 | 0.3–2.9 | 0.952 | 1.3 | 0.5–3.6 | 0.625 |
| 5-protein classifier (high vs. low) | 5.8 | 0.8–39.5 | 0.073 | 1.0 | 1.02–1.06 | 0.0008 |

**^*^** Model 1: classifier was dichotomized as high vs low, using median split 8.3

**^**^** Model 2: classifier was modeled as a continuous factor

**Table S18.** Comparison of performance of commercial prostate cancer tests.

| **Test** | **Biomarker Source** | **Assay Targets** | **Clinical Endpoint** | **Results** | **References^*^** |
| --- | --- | --- | --- | --- | --- |
| Oncotype DX, Genomic Health | Biopsy Very low and low NCCN risk | 12+5 RNA targets (four pathways) | Predicts adverse pathology, MET | AUC = 0.73 combined with CAPRA-S or NCCN | Klein et al, Eur Urol, 2014; Cullen et al, Eur Urol, 2015 |
| Prolaris, Myriad Genetics | Biopsy Prostatectomy Very low and low NCCN risk | 31+15 RNA targets (cell cycle pathway) | Predicts prostate specific death, MET | Biopsy: HR = 1.65 Prostatectomy: HR = 1.77 | Cuzick et al, Lancet Oncol, 2011; Cooperberg et al, J Clin Oncol, 2013 |
| Decipher, GenomeDX | Prostatectomy | 22 RNA targets (multiple pathways) | Predicts adverse pathology, MET | AUC = 0.75–0.79 combined with CAPRA-S or NCCN | Karnes et al, J Urol, 2013 (AUC = 0.79); Spratt et al, J Clin Oncol, 2017 (C-Index = 0.81);  Spratt et al, J Clin Onc, 2018 (C-Index = 0.84) |
| 5-protein classifier | Prostatectomy | 5 proteins | Predicts adverse pathology, MET | HR=5.09  AUC=0.92 combined with age, race and NCCN | Current study |

^*^ References:

1. Klein, E.E.; Cooperberg, M.R.; Magi-Galluzzi, C.; Simko, J.P.; Falzarano, S.M.; Maddala, T.; Chan, J.M.; Li, J.; Cowan, J.E.; Tsiatis, A.C.; et al. A 17-gene Assay to Predict Prostate Cancer Aggressiveness in the Context of Gleason Grade Heterogeneity, Tumor Multifocality, and Biopsy Undersampling. *Eur. Urol.* **2014**, *66*, 550–560.
2. Cullen, J.; Rosner, I.L.; Brand, T.C.; Zhang, N.; Tsiatis, A.C.; Moncur, J.; Ali, A.; Chen, Y.; Knezevic, D.; Maddala, T.; et al. A Biopsy-based 17-gene Genomic Prostate Score Predicts Recurrence After Radical Prostatectomy and Adverse Surgical Pathology in a Racially Diverse Population of Men with Clinically Low- and Intermediate-risk Prostate Cancer. *Eur. Urol.* **2015**, *68*, 123–131.
3. Cuzick, J.; Swanson, G.P.; Fisher, G.; Brothman, A.R.; Berney, D.; Reid, J.E.; Mesher, D.; Speights, V.; Stankiewicz, E.; Foster, C.S.; et al. Prognostic value of an RNA expression signature derived from cell cycle proliferation genes in patients with prostate cancer: a retrospective study. *Lancet Oncol.* **2011**, *12*, 245–55.
4. Cooperberg, M.R.; Simko, J.P.; Cowan, J.E.; Reid, J.E.; Djalilvand, A.; Bhatnagar, S.; Gutin, A.; Lanchbury, J.S.; Swanson, G.P.; Stone, S.; et al. Validation of a Cell-Cycle Progression Gene Panel to Improve Risk Stratification in a Contemporary Prostatectomy Cohort. *J. Clin. Oncol.* **2013**, *31*, 1428–1434.
5. Karnes, R.J.; Bergstralh, E.J.; Davicioni, E.; Ghadessi, M.; Buerki, C.; Mitra, A.P.; Crisan, A.; Erho, N.; Vergara, I.A.; Lam, L.L.; et al. Validation of a genomic classifier that predicts metastasis following radical prostatectomy in an at risk patient population. *J. Urol.* **2013**, *190*, 2047–2053.
6. Spratt, D.E.; Yousefi, K.; Deheshi, S.; Ross, A.E.; Den, R.B.; Schaeffer, E.M.; Trock, B.J.; Zhang, J.; Glass, A.G.; Dicker, A.P.; et al. Individual Patient-Level Meta-Analysis of the Performance of the Decipher Genomic Classifier in High-Risk Men After Prostatectomy to Predict Development of Metastatic Disease. *J. Clin. Oncol.* **2017**, *35*, 1991–1998.
7. Spratt, D.E.; Zhang, J.; Santiago-Jiménez, M.; Dess, R.T.; Davis, J.W.; Den, R.B.; Dicker, A.P.; Kane, C.J.; Pollack, A.; Stoyanova, R.; et al. Development and Validation of a Novel Integrated Clinical-Genomic Risk Group Classification for Localized Prostate Cancer. *J. Clin. Oncol.* **2018**, *36*, 581–590.
